# Redirecting photosynthetic electron flux in the cyanobacterium *Synechocystis* sp. PCC 6803 by the deletion of flavodiiron protein Flv3

**DOI:** 10.1186/s12934-019-1238-2

**Published:** 2019-11-05

**Authors:** Kati Thiel, Pekka Patrikainen, Csaba Nagy, Duncan Fitzpatrick, Nicolas Pope, Eva-Mari Aro, Pauli Kallio

**Affiliations:** 1 Molecular Plant Biology, Department of Biochemistry, University of Turku, 20014 Turun Yliopisto, Finland; 2 Department of Future Technologies, University of Turku, 20014 Turun Yliopisto, Finland; 3 Itäinen Pitkäkatu 4 C, 20520 Turku, Finland

**Keywords:** *Synechocystis* sp., PCC 6803, Flavodiiron protein 3 (Flv3), Cyanobacterial engineering, Sucrose, Polyhydroxybutyrate, Membrane Inlet Mass Spectrometry

## Abstract

**Background:**

Oxygen-evolving photoautotrophic organisms, like cyanobacteria, protect their photosynthetic machinery by a number of regulatory mechanisms, including alternative electron transfer pathways. Despite the importance in modulating the electron flux distribution between the photosystems, alternative electron transfer routes may compete with the solar-driven production of CO_2_-derived target chemicals in biotechnological systems under development. This work focused on engineered cyanobacterial *Synechocystis* sp. PCC 6803 strains, to explore possibilities to rescue excited electrons that would normally be lost to molecular oxygen by an alternative acceptor flavodiiron protein Flv1/3—an enzyme that is natively associated with transfer of electrons from PSI to O_2_, as part of an acclimation strategy towards varying environmental conditions.

**Results:**

The effects of Flv1/3 inactivation by *flv3* deletion were studied in respect to three alternative end-products, sucrose, polyhydroxybutyrate and glycogen, while the photosynthetic gas fluxes were monitored by Membrane Inlet Mass Spectrometry (MIMS) to acquire information on cellular carbon uptake, and the production and consumption of O_2_. The results demonstrated that a significant proportion of the excited electrons derived from photosynthetic water cleavage was lost to molecular oxygen via Flv1/3 in cells grown under high CO_2_, especially under high light intensities. In *flv3* deletion strains these electrons could be re-routed to increase the relative metabolic flux towards the monitored target products, but the carbon distribution and the overall efficiency were determined by the light conditions and the genetic composition of the respective pathways. At the same time, the total photosynthetic capacity of the Δ*flv3* strains was systematically reduced, and accompanied by upregulation of oxidative glycolytic metabolism in respect to controls with the native Flv1/3 background.

**Conclusions:**

The observed metabolic changes and respective production profiles were proposedly linked with the lack of Flv1/3-mediated electron transfer, and the associated decrease in the intracellular ATP/NADPH ratio, which is bound to affect the metabolic carbon partitioning in the *flv3*-deficient cells. While the deletion of *flv3* could offer a strategy for enhancing the photosynthetic production of desired chemicals in cyanobacteria under specified conditions, the engineered target pathways have to be carefully selected to align with the intracellular redox balance of the cells.

## Introduction

Photosynthetic cyanobacteria have been considered as potential next-generation biotechnological hosts for the production of different carbon-based products, as part of the development of new carbon neutral industrial solutions to replace petroleum-derived chemicals now in use [1, 2]. The critical advantage of such autotrophic production platforms would be the possibility to convert CO_2_ directly into the target metabolites by using photosynthetically captured solar radiation as the sole energy source, and cyanobacterial cells as biological catalysts. Importantly, this would decouple the production process from the dependence of biomass-based substrates, which is a key limitation in all currently existing large-scale biotechnological applications that use heterotrophic organisms as hosts. Although cyanobacteria have been engineered to produce a wide range of different chemicals [3–6], the development of commercially feasible technologies is still restricted by inadequate efficiency at which the solar energy harvested by the cells can be captured in the desired target products. The challenge, from biological engineering perspective, is that without extensive understanding of interactions between solar energy conversion and subsequent carbon reduction metabolism, the harvested energy is distributed to a number of cellular functions and easily lost in unwanted reactions, which compete with the pathways of interest.

**Fig. 1:**
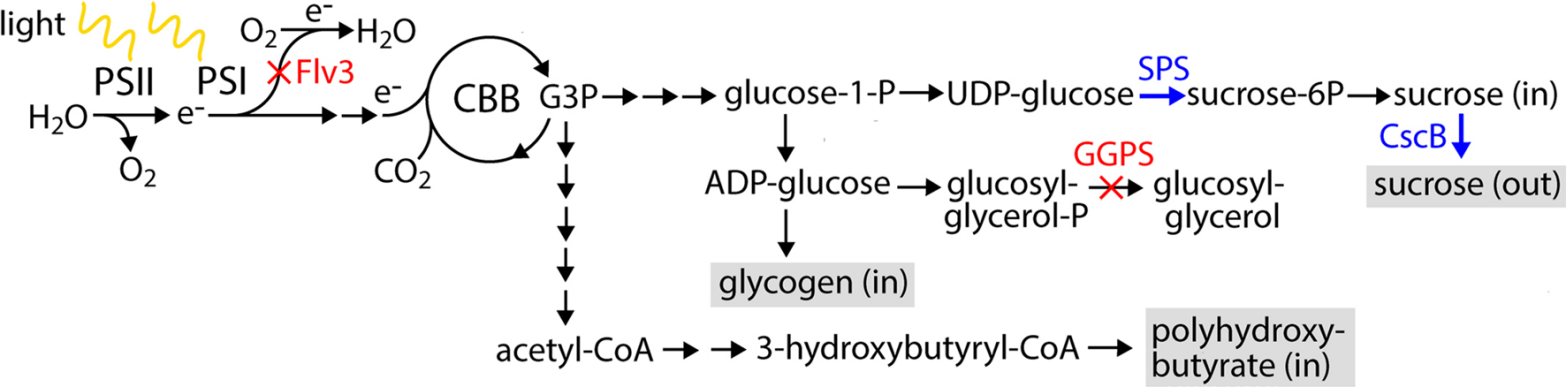
Simplified representation of pathways engineered in *Synechocystis* to evaluate the possibility to enhance the photosynthetic electron flux to target products by the inactivation of the flavodiiron protein 3 (Flv3) and improving the strength of the electron sink (see Table 1 for strain descriptions). The engineering strategy made use of the native capacity of cyanobacterial cells to alleviate osmotic stress by the production of intracellular sucrose and glucosylglycerol as osmoprotective agents. Specific genetic modifications introduced in *Synechocystis* to maximize the production of sucrose include (i) the inactivation (red) of Flavodiiron protein 3 (Flv3 encoded by *sll0550*) which is involved in a photoprotective Mehler-like reaction of the photosynthetic electron transfer chain i.e. the loss of excited electrons to molecular oxygen, and (ii) the overexpression (blue) of sucrose permease (CscB from *E. coli*) responsible for the active transport of sucrose out from the cell into the culture medium. In addition, the modifications include (iii) the inactivation (red) of glucosylglycerolphosphate synthase (ggpS encoded by *sll1566*) responsible for a committed step in the biosynthesis of glucosylglycerol, competing with the sucrose biosynthesis pathway, and (iv) the overexpression (blue) of sucrose phosphate synthase (SPS encoded by *sll0045*) which enhances one of the potentially limiting steps, conversion of UDP-glucose to sucrose-6-phosphate. The target product sucrose and the storage compounds, polyhydroxybutyrate and glycogen, quantitated in the study are shown in grey background. (See Additional file 1: Table S2 for a more comprehensive list of the enzymatic reactions in the pathways, and the production and use of ATP and NADPH)

The objective of the study was to investigate whether the autotrophic production efficiency of specified end-metabolites can be improved in engineered cyanobacterial cells by the modification of endogenous regulatory systems, which are involved in the redistribution of photosynthetic electron fluxes when the light reactions and carbon fixation are not in balance. The focus was on a specific heterodimeric cyanobacterial Class C flavodiiron protein Flv1/3 (see review [7]), which directs electrons accumulated in the photosynthetic electron transfer chain via photosystem (PS) I to molecular oxygen. In this so-called Mehler-like reaction [8, 9], Flv1/3 uses electrons which originate from the photosynthetic water-splitting in PSII and are thereafter directed via PSI to reduce O_2_ to water (Fig. 1), without the formation of ROS [10]. This reaction has been shown to be particularly important for preventing the damage to PSI, especially under growth conditions such as fluctuating light that increase the reductive pressure inflicted on PSI [9, 11, 12]. Flv1/3-mediated O_2_ photoreduction has also been observed in cells cultured under continuous light [8, 10], as well as in the presence of saturating carbon where it has been reported to account for the loss of ~ 20% of the electrons originating from PSII [8], although under these conditions the enzyme is not essential for viability. Notably, the channeling of electrons to O_2_ by Flv1/3 instead of NADP^+^, is bound to increase the intracellular ATP/NADPH ratio, thus potentially linking to a range of effects associated with the metabolic state of the cell.

The leading question here was whether the elimination of the native Flv1/3 reaction could be used to enhance the photosynthetic electron flux towards alternative carbon sinks in engineered cyanobacterial cells, thus improving the yield of target end-products without compromising the fitness of the host. The strategy was to inactivate the gene coding for Flv3 (*sll0550*) to suppress the oxygen-dependent Flv1/3 Mehler-like activity [8, 9] in the cyanobacterium *Synechocystis* sp. PCC 6803 (*Synechocystis* from here on), followed by the analysis of the consequent effects on specific pathway flux distributions, cell growth, and photosynthetic gas fluxes. The experimental set-up was limited specifically to elevated carbon atmosphere, as to represent conditions where additional CO_2_ is provided from available point sources, with the aim of assessing the engineering potential and obtaining new biological insight into the metabolic interactions involving Flv3.

## Results

### Sucrose as a reporter compound in engineered Δ*flv3* strains

**Fig. 2:**
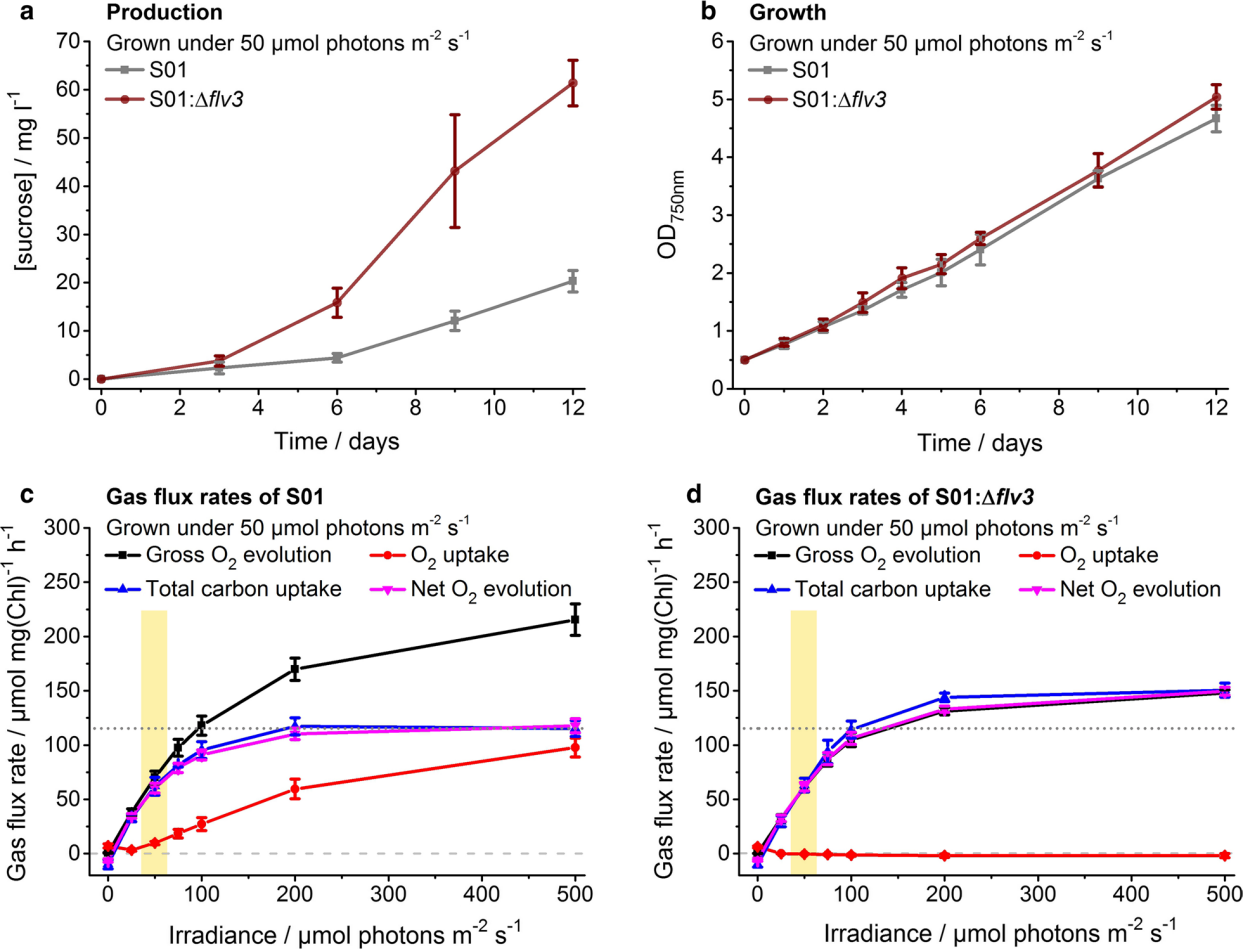
Characterization of engineered sucrose-producing *Synechocystis* strains grown under continuous 50 μmol photons m^−2^ s^−1^ light. The strains S01 (over-expression of sucrose permease CscB) and S01:Δ*flv3* (over-expression of CscB and inactivation of flavodiiron protein Flv3) were evaluated in respect to (**a**) sucrose production (mg l^−1^), (**b**) growth (OD_750nm_) and (**c**, **d**) gas flux rates. In **a** and **b**, the control strain S01 is shown in grey and the strain S01:Δ*flv3* in brown. In **c** and **d**, the lines represent the gross O_2_ evolution (black), O_2_ uptake (red), total carbon uptake (blue) and net O_2_ evolution (magenta) measured on day 5 as a function of irradiance for the strains S01 and S01:Δ*flv3*, respectively. The irradiance corresponding to the growth light intensity is highlighted in yellow. To assist the comparison between **c** and **d**, the zero gas flux level and the S01 control strain total carbon uptake level at 500 μmol photons m^−2^ s^−1^ have been shown in horizontal dashed line and dotted line, respectively (see Additional file 1: Table S1 for calculated statistical comparison). The strains were cultivated in 1% CO_2_ in the presence of 400 mM supplemented NaCl. In each case, the average and standard deviation were calculated from three to four independent experiments

**Fig. 3:**
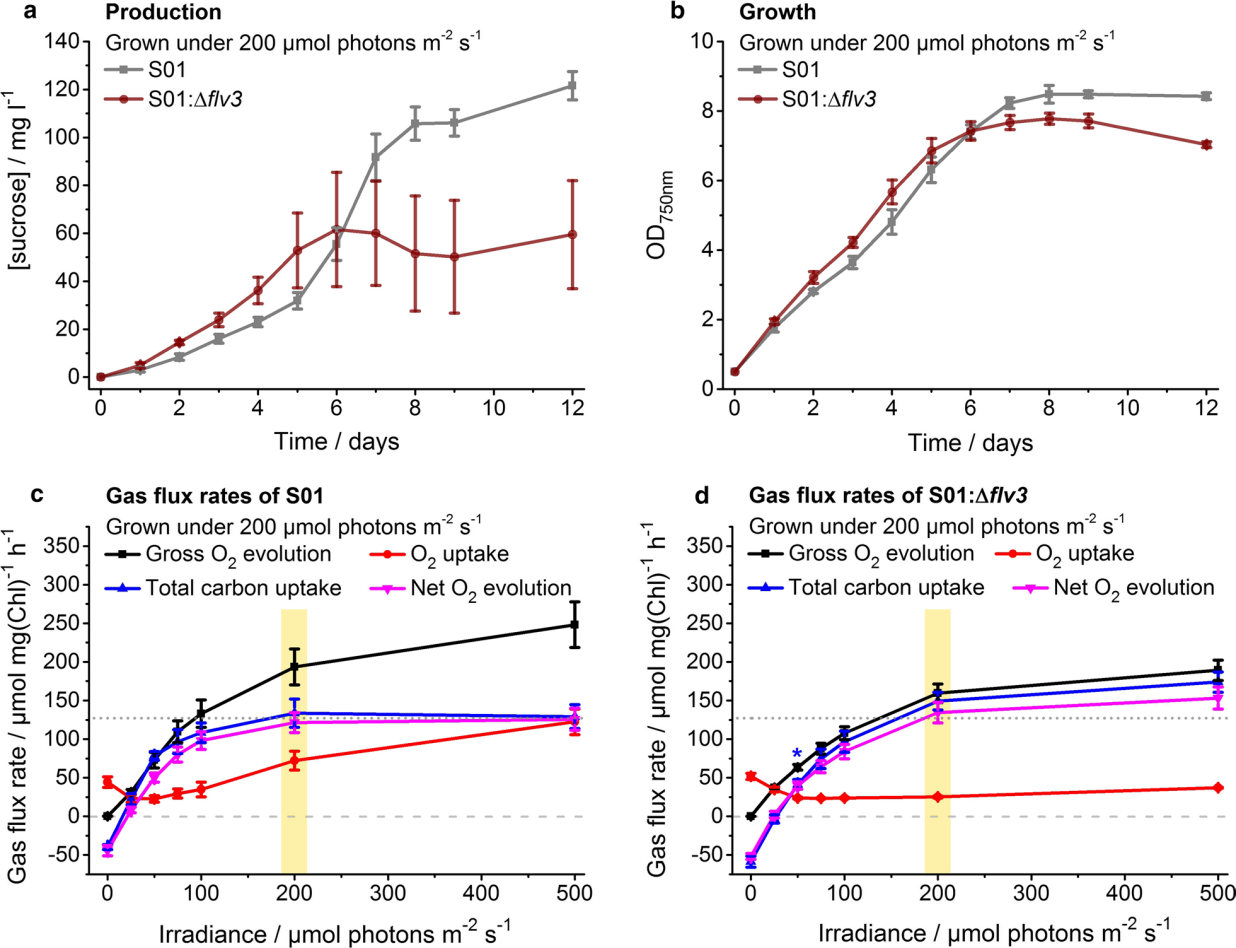
Characterization of engineered sucrose-producing *Synechocystis* strains grown under continuous 200 μmol photons m^−2^ s^−1^ light. The strains S01 (over-expression of sucrose permease CscB) and S01:Δ*flv3* (over-expression of CscB and inactivation of flavodiiron protein Flv3) were evaluated in respect to (**a**) sucrose production (mg l^−1^) and (**b**) growth (OD_750nm_) and (**c**, **d**) gas flux rates. In **a** and **b**, the control strain S01 is shown in grey and the strain S01:Δ*flv3* in brown. In **c** and **d**, the lines represent the gross O_2_ evolution (black), O_2_ uptake (red), total carbon uptake (blue) and net O_2_ evolution (magenta) measured on day 5 as a function of irradiance for the strains S01 and S01:Δ*flv3*, respectively. The irradiance corresponding to the growth light intensity is highlighted in yellow. To assist the comparison between **c** and **d**, the zero gas flux level and the S01 control strain total carbon uptake level at 500 μmol photons m^−2^ s^−1^ have been shown in horizontal dashed line and dotted line, respectively (see Additional file 1: Table S1 for calculated statistical comparison). The strains were cultivated in 1% CO_2_ in the presence of 400 mM supplemented NaCl. In each case, the average and standard deviation were calculated from three to four independent experiments

**Fig. 4:**
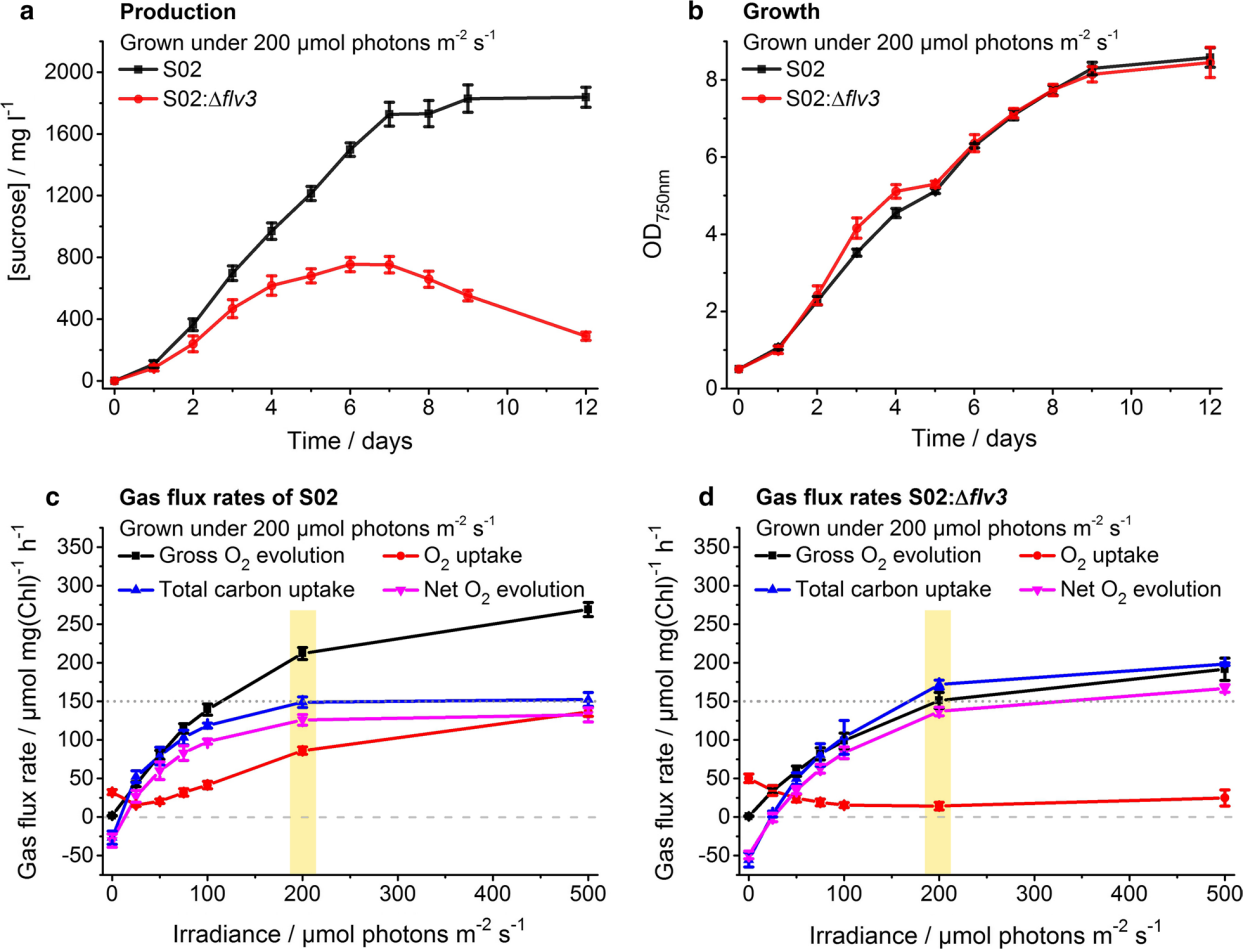
Characterization of engineered sucrose-producing *Synechocystis* strains grown under continuous 200 μmol photons m^−2^ s^−1^ light. The strains S02 (over-expression of sucrose permease CscB and sucrose phosphate synthase SPS, and inactivation of glucosylglyserolphosphate synthase GGPS) and S02:Δ*flv3* (over-expression of CscB and SPS, and inactivation of GGPS and flavodiiron protein Flv3) were evaluated in respect to (**a**) sucrose production (mg l^−1^), (**b**) growth (OD_750nm_) and (**c**, **d**) gas flux rates. In **a** and **b**, the control strain S02 is shown in black and the strain S02:Δ*flv3* in red. In **c** and **d**, the lines represent the gross O_2_ evolution (black), O_2_ uptake (red), total carbon uptake (blue) and net O_2_ evolution (magenta) measured on day 5 as a function of irradiance for the strains S02 and S02:Δ*flv3*, respectively. The irradiance corresponding to the growth light intensity is highlighted in yellow. To assist the comparison between **c** and **d**, the zero gas flux level and the S02 control strain total carbon uptake level at 500 μmol photons m^−2^ s^−1^ have been shown in horizontal dashed line and dotted line, respectively (see Additional file 1: Table S1 for calculated statistical comparison). The strains were cultivated in 1% CO_2_ in the presence of 400 mM supplemented NaCl. In each case, the average and standard deviation were calculated from three to four independent experiments

**Fig. 5:**
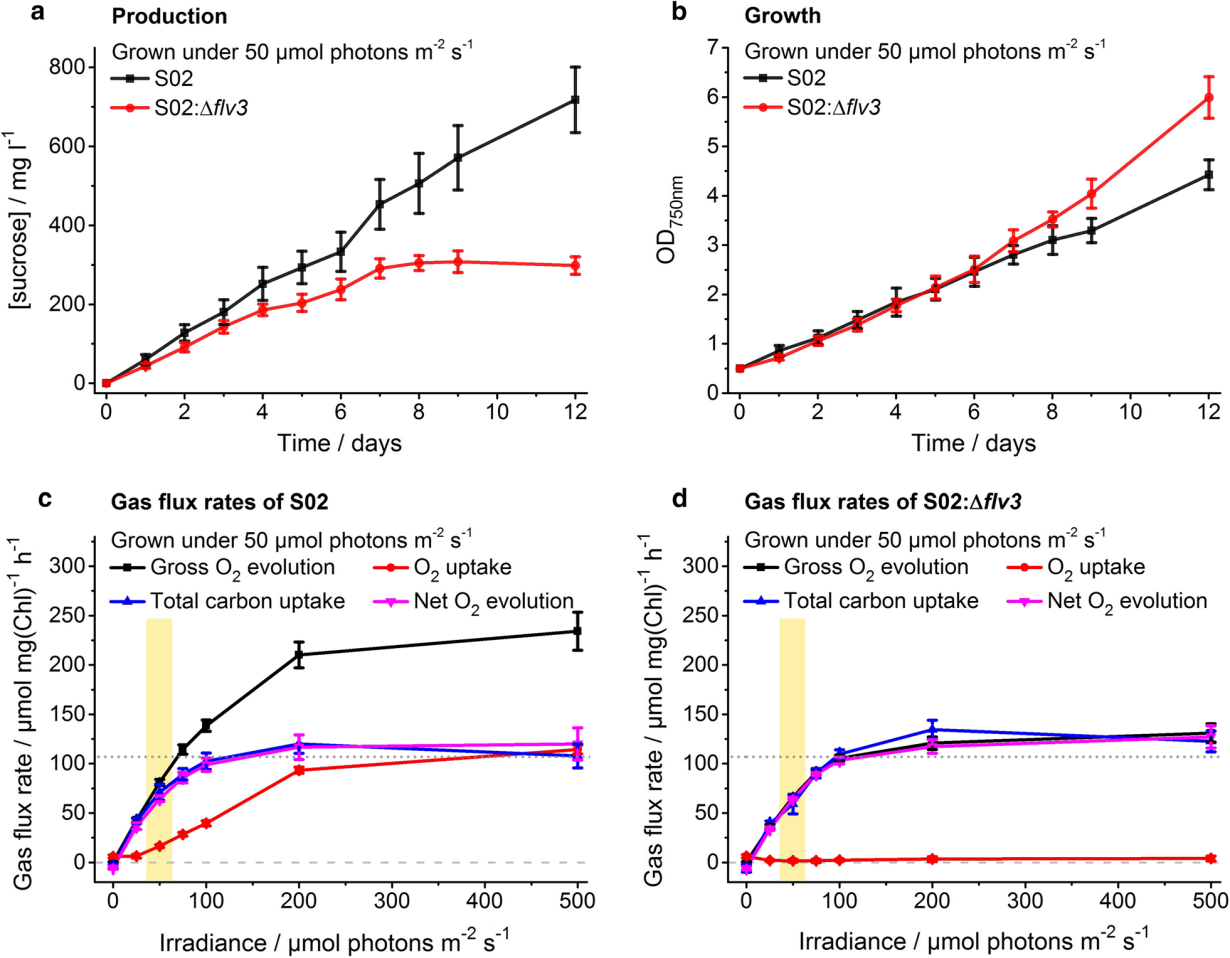
Characterization of engineered sucrose-producing *Synechocystis* strains grown under continuous 50 μmol photons m^−2^ s^−1^ light. The strains S02 (over-expression of sucrose permease CscB and sucrose phosphate synthase SPS, and inactivation of glucosylglyserolphosphate synthase GGPS) and S02:Δ*flv3* (over-expression of CscB and SPS, and inactivation of GGPS and flavodiiron protein Flv3) were evaluated in respect to (**a**) sucrose production (mg l^−1^), (**b**) growth (OD_750nm_) and (**c**, **d**) gas flux rates. In **a** and **b**, the control strain S02 is shown in black and the strain S02:Δ*flv3* in red. In **c** and **d**, the lines represent the gross O_2_ evolution (black), O_2_ uptake (red), total carbon uptake (blue) and net O_2_ evolution (magenta) measured on day 5 as a function of irradiance for the strains S02 and S02:Δ*flv3*, respectively. The irradiance corresponding to the growth light intensity is highlighted in yellow. To assist the comparison between **c** and **d**, the zero gas flux level and the SO2 control strain total carbon uptake level at 500 μmol photons m^−2^ s^−1^ have been shown in horizontal dashed line and dotted line, respectively (see Additional file 1: Table S1 for calculated statistical comparison). The strains were cultivated in 1% CO_2_ in the presence of 400 mM supplemented NaCl. In each case, the average and standard deviation were calculated from three to four independent experiments

To evaluate possible effects of *flv3* inactivation on autotrophic production efficiency in the cyanobacterial host *Synechocystis*, *sucrose* was selected as a reporter compound, and a generic representative of target chemicals derived from CO_2_ through pathway engineering. Besides low toxicity and available heterologous transporter which allows excretion into the extracellular matrix, sucrose was initially considered as a potential candidate because of the relatively high reported yields in comparison to other compounds [13–15]. In addition, a number of genetic modifications which facilitate sucrose biosynthesis have been previously studied [14, 15], providing access to different pathway variations (Fig. 1). The concept is based on the native property of *Synechocystis* to produce sucrose as an intracellular osmoprotectant when exposed to high external ion concentrations, in combination with the introduction of a heterologous *sucrose permease* (CscB from *Escherichia coli*), which transports the sucrose out from the cell [14]. The sucrose permease expression mutant S01 (Table 1) was set as the starting point, and served as the background for every strain constructed in the work. Following this strategy, two different variations of *Synechocystis flv3* deletion strains (Table 1; strains S01:Δ*flv3* and S02:Δ*flv3*) and three control strains (Table 1; strains S01, S01:compl and S02) were designed and assembled (Additional file 1: Fig. S1) (see Fig. 1 for functional information on the associated proteins). When cultivated in the presence of 400 mM NaCl, the generated strains were shown to produce and accumulate sucrose in the culture medium (Figs. 2a, 3a, 4a, 5a) (Additional file 1: Fig. S2a), thus enabling quantitative comparison of the strains with and without Flv1/3.

**Table 1:** The sucrose-producing *Synechocystis* strains generated and compared in this study

Strain ID	Genetic background	OE target(s)	Expression plasmid
S01	Wild-type	CscB	pDF-lac2-cscB-CmR
S01:Δ*flv3*	Δ*flv3*	CscB	pDF-lac2-cscB-CmR
S01:compl	Δ*flv3*	CscB + Flv3	pDF-lac2-cscB-flv3-CmR
S02	Δ*ggpS*	CscB + SPS	pDF-lac2-cscB-sps-CmR
S02:Δ*flv3*	Δ*flv3 *+ Δ*ggpS*	CscB + SPS	pDF-lac2-cscB-sps-CmR

The enzymes targeted for inactivation and over-expression are Flv3 (flavodiiron protein 3 from *Synechocystis*), GGPS (glucosylglycerolphosphate synthase from *Synechocystis*), CscB (sucrose permease from *E. coli*), and SPS (sucrose phosphate synthase from *Synechocystis*)

### Inactivation of *flv3* enhances sucrose production under low light conditions

Although the absolute sucrose productivities were low, the deletion of *flv3* in *Synechocystis* (Table 1; strain S01:Δ*flv3*) resulted in about three-fold increase in total sucrose accumulation over a 12-day cultivation period in the presence of 1% CO_2_ under moderate light (50 µmol photons m^−2^ s^−1^) (Fig. 2a) and six-fold increase under low growth light (20 µmol photons m^−2^ s^−1^) (Additional file 1: Fig. S2a) in comparison to the control strain (Table 1; strain S01). At the same time, the S01:Δ*flv3* mutant and the S01 control strain could not be distinguished from one another based on growth (Fig. 2b) (Additional file 1: Fig. S2b) or the pigment profile (Additional file 1: Fig. S3), implying that under these conditions the absence of Flv3 did not inflict any significant metabolic stress to the cell. Parallel analysis of the cellular gas fluxes by Membrane Inlet Mass Spectrometry (MIMS) showed that upon short-term exposure to increasing light intensities, the light-dependent oxygen uptake characteristic for the S01 control strain with intact Flv1/3 (Fig. 2c) was not observed in the S01:Δ*flv3* mutant (Fig. 2d). In addition, the net oxygen evolution and total carbon uptake appeared to be systematically upregulated in the S01:Δ*flv3* deletion strain (Fig. 2c, d) (Additional file 1: Table S1). Even though the changes could not be directly quantitatively correlated with the enhanced sucrose productivity measured under long-term cultivation in low constant light, the analysis demonstrated the *potential* for taking advantage of the surplus electrons natively lost in the Flv1/3 reaction for O_2_ reduction. In order to further corroborate the established link between the observed effects and the absence of native Flv1/3 heterodimer in the mutant, an over-expression construct for Flv3 was introduced into the S01:Δ*flv3* strain (Table 1; strain S01:compl). Accordingly, this resulted in partial complementation of the effects induced by *flv3* inactivation, as seen in the suppression of sucrose accumulation towards the S01 control strain levels (Additional file 1: Fig. S4a), and re-established O_2_ uptake in light (Additional file 1: Fig. S4b) characteristic to the background with native Flv1/3.

### The Δ*flv3*-induced effects are dependent on both the light intensity and the genetic context

In order to evaluate the effect of *flv3* deletion in response to increased photosynthetic electron flux, the generated sucrose-producing *Synechocystis* strains (Table 1; strains S01 and S01:Δ*flv3*) were next cultivated and compared under higher growth light intensity (200 µmol photons m^−2^ s^−1^). Over the first five days the S01:Δ*flv3* strain showed slight enhancement in sucrose production (Fig. 3a) and in growth (Fig. 3b) in comparison to the corresponding S01 control. As before, the light-induced oxygen uptake of the S01:Δ*flv3* strain was clearly decreased, and the total carbon uptake and net O_2_ production (measured at higher irradiancies) were increased in reference to S01 (Fig. 3c, d) (Additional file 1: Table S1). However, unlike in lower growth light, the relative productivity (Fig. 3a) and growth of the *flv3* deletion strain (Fig. 3b) declined after the cultivation day six, implicating that the fitness of the cells lacking Flv1/3 may have been compromised when approaching higher culture densities under 200 µmol photons m^−2^ s^−1^. To further assess the impact of increasing the electron sink capacity, additional modifications were introduced into the host with the objective to reinforce the flux towards sucrose. These modifications included the over-expression of *sucrosephosphate synthase* (SPS; encoded by *sll0045*) which has been shown to be one of the limiting steps in sucrose biosynthesis [15], as well as the inactivation of *glucosylglycerolphosphate synthase* (GGPS; encoded by *sll1566*) essential for the formation of a parallel osmoprotectant glucosylglycerol in *Synechocystis* [15] (Fig. 1) (Table 1; strains S02 and S02:Δ*flv3*). In comparison to the original engineered strains (Table 1; strains S01 and S01:Δ*flv3*, Fig. 3), these auxiliary modifications dramatically enhanced the overall sucrose productivity at 200 µmol photons m^−2^ s^−1^ (Table 2, Fig. 4). However, the control S02 strain harboring the native *flv3* gene (Table 1; strain S02) accumulated sucrose at a significantly higher rate (~ 273 vs. ~ 183 mg l^−1^ day^−1^) (Table 2) and to a higher final maximum concentration (~ 1800 vs. ~ 750 mg l^−1^) (Fig. 4a) than the S02:Δ*flv3* strain (Table 1; strain S02:Δ*flv3*). This difference in production efficiency appeared not to be caused by reduced fitness of the Δ*flv3* cells, as the absence of Flv1/3 under these conditions did not have any apparent negative effect on the growth of the strain (Fig. 4b).

**Table 2:** Sucrose production rates of engineered *Synechocystis* strains grown under 1% CO_2_ in the presence of 400 mM supplemented NaCl under different light intensities

Growth light/μmol photons m^−2^ s^−1^	Strain	Production rate ± std/mg l^−1^ day^−1^	Time period/days	Figure number
20	S01	0.54 ± 0.19	3–12	Additional file 1: Fig. S2
	S01:Δ*flv3*	3.22 ± 0.46	3–12	Additional file 1: Fig. S2
50	S01	2.65 ± 0.05	6–12	Fig. 2
	S01:Δ*flv3*	7.62 ± 0.35	6–12	Fig. 2
	S02	59.08 ± 2.61	1–5	Fig. 5
	S02:Δ*flv3*	47.65 ± 1.20	1–4	Fig. 5
200	S01	29.91 ± 3.64	5–7	Fig. 3
	S01:Δ*flv3*	11.76 ± 0.88	1–5	Fig. 3
	S02	273.02 ± 5.33	1–7	Fig. 4
	S02:Δ*flv3*	183.03 ± 10.69	1–4	Fig. 4

Production rates are calculated from the sucrose production experiments shown in Figs. 2, 3, 4, 5 and Additional file 1: Fig. S2 between the days indicated in table

### Measured gas exchange rates support relative increase in biosynthetic potential in the S02 *flv3* deletion strain

To obtain a more comprehensive view of the overall impact caused by the *flv3* deletion, also the enhanced sucrose production strains S02 and S02:Δ*flv3* (Table 1) were subjected to gas-flux analysis by MIMS under increasing light intensities. As initially observed for the S01 strains (Figs. 2c, d, 3c, d) (Additional file 1: Table S1), the light-induced O_2_ uptake of the S02:Δ*flv3* mutant grown at 200 µmol photons m^−2^ s^−1^ was significantly decreased in comparison to the control strain (Fig. 4c, d) (Additional file 1: Table S1). The S02:Δ*flv3* strain also exhibited higher net O_2_ evolution and total carbon uptake capacity (Fig. 4d) (Additional file 1: Table S1) than the control strain S02 (Fig. 4c). These findings demonstrated that the increase in the availability of reducing equivalents in the S02:Δ*flv3* mutant could potentially be used to enhance CO_2_ fixation, and thus to advance the downstream metabolic processes, although the carbon in this case appeared not to be channeled to sucrose production (Fig. 4a) or cell proliferation (Fig. 4b). A similar pattern in sucrose accumulation (Fig. 5a), cell growth (Fig. 5b) and gas-fluxes (Fig. 5c, d) (Additional file 1: Table S1) was recorded also for the cells grown under moderate light intensity (50 µmol photons m^−2^ s^−1^). Importantly, however, the gross O_2_ evolution—a direct measure of the PSII activity as the method distinguishes between oxygen consumption and production—was markedly lowered in the S02:Δ*flv3* mutant throughout the profile (Figs. 4c, d, 5c, d) (Additional file 1: Table S1). Also observed to a lower degree in the S01 strains (Figs. 2c, d, 3c, d) (Additional file 1: Table S1), this implied that the overall photosynthetic capacity may have been compromised in the absence of Flv1/3. These findings emphasized that the specific light conditions (50 µmol photons m^−2^ s^−1^ versus 200 µmol photons m^−2^ s^−1^), and the genetic composition of the engineered pathways (i.e. combination of the over-expression and knock-out targets), together dictated the performance of the engineered Δ*flv3* strains in respect to photosynthetic activity and sucrose productivity.

### The deletion of *flv3* results in significantly increased respiration rates

The recorded sucrose curves very implicitly demonstrated that both ∆*flv3* strains (S01:Δ*flv3* and S02:Δ*flv3*) also *consumed* sucrose. This was clearly seen in the decrease of sucrose concentration measured from the medium of the *flv3* deletion strains, especially in the cultures grown under higher light 200 µmol photons m^−2^ s^−1^ (Figs. 3a, 4a). While it is not possible to estimate the corresponding rates based on these curves, as they represent the combined effect of simultaneous sucrose production and consumption, it was obvious that for the ∆*flv3* strains the uptake of sucrose was faster than its secretion. In comparison, this was not observed for the control strains with intact *flv3* **(**Figs. 3a, 4a), or in cultures grown under lower 50 µmol photons m^−2^ s^−1^ light (Figs. 2a, 5a) (Additional file 1: Fig. S4a). This indicated that the sucrose-producing ∆*flv3* strains grown under constant high light exhibited more profound use of external carbohydrates, which was expected to correspond to increased aerobic respiratory metabolism in the cells. Supporting this finding, while the O_2_ uptake of the ∆*flv3* strains was always low comparison to the control strains expressing Flv1/3, there was a clear correlation between the observed decrease of sucrose in the growth medium and elevated levels of oxygen consumption measured for the ∆*flv3* strains grown under 200 µmol photons m^−2^ s^−1^ (Figs. 3d, 4d), which was not seen in cultures incubated at 50 µmol photons m^−2^ s^−1^ (Figs. 2d, 5d). In addition, the dark respiration rate measured by MIMS was clearly higher for the S02:Δ*flv3* strain (Fig. 4d) than in the corresponding control S02 (Fig. 4c) cultivated under 200 µmol photons m^−2^ s^−1^, and markedly increased (up to five-fold) in comparison to all the strains grown under low light (Figs. 2, 5).

### Polyhydroxybutyrate and glycogen serve as alternative electron sinks in the Δ*flv3* mutant

**Fig. 6:**
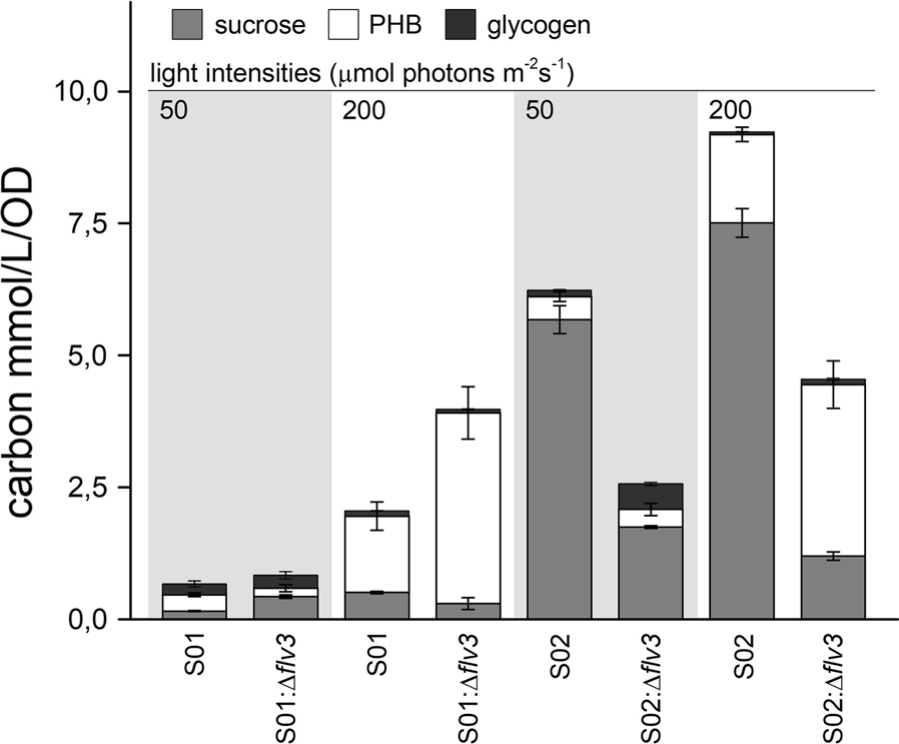
Molar comparison of carbon allocated for the production of sucrose, PHB and glycogen by the engineered *Synechocystis* strains (Table 1) grown under 50 and 200 μmol photons m^−2^ s^−1^ light at the cultivation day 12. The values have been calculated from the data shown in Figs. 2, 3, 4, 5 (sucrose), Additional file 1: Fig. S5 (PHB) and Additional file 1: Fig. S6 (glycogen)

To further assess possible differences in the metabolic flux and carbon allocation between the two *flv3* deletion mutants (S01:Δ*flv3* and S02:Δ*flv3*) and the control strains (S01 and S02), we quantitated the intracellular storage compounds, polyhydroxybutyrate (PHB) (Additional file 1: Fig. S5) and glycogen (Additional file 1: Fig. S6), in cells grown in the presence of 1% CO_2_ under moderate-light and high-light. The most profound change was observed in the PHB content of the Δ*flv3* strains (Table 1; S01:Δ*flv3* and S02:Δ*flv3*) cultured under 200 µmol photons m^−2^ s^−1^ (Additional file 1: Fig. S5): Out of the three analyzed end-products, PHB was now clearly the primary carbon sink in the cells lacking *flv3* (Fig. 6; white bar), and the amount was about double in comparison to the corresponding control strains S01 and S02 by the cultivation day 12. Notably, this effect was not observed in cells cultured under low light 50 µmol photons m^−2^ s^−1^ (Fig. 6) (Additional file 1: Fig. S5). As for the total carbon allocation, the overall role of glycogen appeared to be less significant than that of sucrose or PHB (Fig. 6). The absence of *flv3* resulted in a clear relative increase in the glycogen content only in S02:Δ*flv3* (i.e. the Δ*ggpS* strain over-expressing SPS) (Table 1) when grown under 50 µmol photons m^−2^ s^−1^ (Additional file 1: Fig. S6c), while in other cases the levels remained comparable to the controls (Fig. 6) (Additional file 1: Fig. S6).

### In silico analysis reveals clear differences between the ATP/NADPH requirement of the alternative product pathways

In order to evaluate the theoretical ATP/NADPH demand of the three alternative pathways, the number of molecules of ATP and/or NADPH consumed or produced in the stepwise enzyme-catalyzed steps starting from the common intermediate G3P towards sucrose, glycogen and PHB, were calculated using the *Synechocystis* metabolic model iSynCJ816 [16] as a template (see Additional file 1: Table S2). Although the criteria did not take into account the side reaction pathways (i.e. the generation of cosubstrates) beyond the first branching node, it provided a descriptive estimate of the biosynthetic cost between the three pathways. Based on the calculation, the sucrose pathway was shown not to consume or produce any ATP or NADPH and is hence neutral towards the prevalent ATP/NADPH equilibrium, while the glycogen pathway consumes one ATP without any net effect on the NADPH quota, thus reducing the intracellular ATP/NADPH ratio. In stark contrast, the PHB pathway produces four molecules of ATP and consumes one molecule of NADPH, and thus may significantly increase the ATP/NADPH ratio. This is an opposite effect to that expected to result from *flv3* deletion, which potentially decreases the ratio by allowing a higher relative electron flux to NADP^+^, instead of O_2_.

### Deletion of *flv3* reduces the intracellular ATP/NADPH ratio in *Synechocystis*

**Fig. 7:**
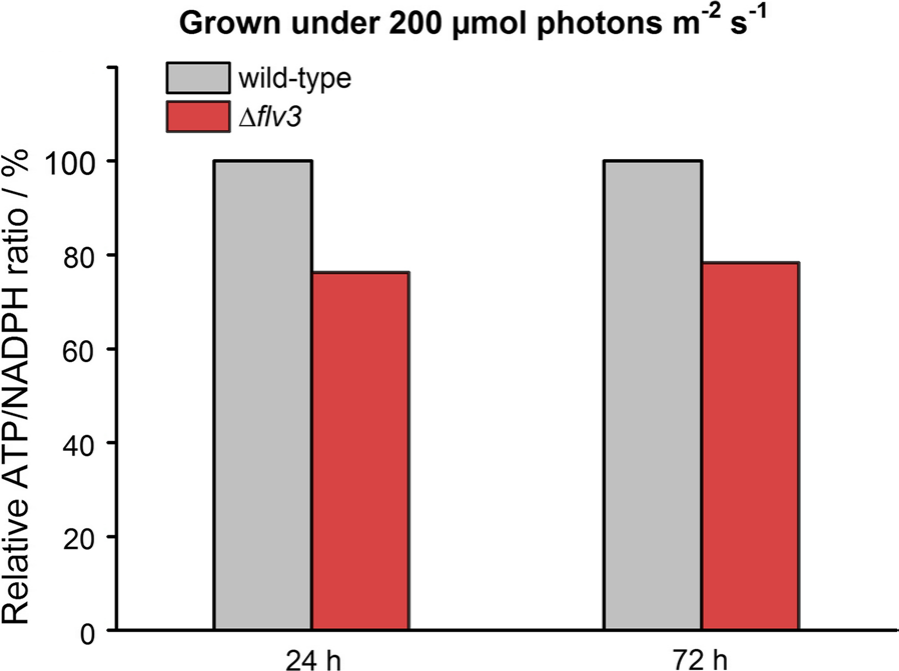
Relative ATP/NADPH ratios of *Synechocystis* wild-type and ∆*flv3* strains after cultivating the cells for 24 h and 72 h under continuous 200 μmol photons m^−2^ s^−1^ light. The values were calculated from the average luminescence data presented in Additional file 1: Fig. S8, and normalized to the wild-type ratio set to represent 100%

To experimentally corroborate the effect of *flv3* deletion on the intracellular ATP/NADPH ratio in *Synechocystis*, the unmodified background strain (WT), and a corresponding ∆*flv3* mutant strain were grown under standard conditions, and analyzed for the amount of ATP and NADPH at two different time points (Additional file 1: Figs. S7, S8). The relative content of both ATP and NADPH were higher in the ∆*flv3* cells in comparison to the WT after the first day of main culture (Additional file 1: Fig. S8a) but leveled over the following two days of cultivation (Additional file 1: Fig. S8b). Calculation of the relative ATP/NADPH ratios from these values showed that the ratio in the ∆*flv3* strain was lower than in the WT, as predicted based on the increase in electron flux to NADP^+^ resulting from the lack of the competing Flv1/3 activity (Fig. 7).

## Discussion

Normal cell metabolism has been optimized in the course of evolution for the allocation of available resources to different functions needed for cell proliferation, maintenance, and acclimation to varying environmental and metabolic cues. However, when microbial cells are recruited as biotechnological production hosts the specific objective is to effectively funnel the metabolic flux towards desired target metabolites, while minimizing the competing reactions and pathways. This means that biomass accumulation and generation of intracellular storage compounds should be avoided at the production phase when aiming at maximal yield of target products with the highest overall cost-efficiency. In the design of applications using cyanobacteria as biotechnological hosts that employ light as the sole energy source for the direct production of chemicals, the pipeline requires efficient coupling of (i) the photosynthetic light reactions, (ii) the subsequent CO_2_ fixation reactions, and (iii) the downstream biosynthetic steps to the end-products excreted into the medium. From this perspective, we focused on the possibilities to enhance the capture of excited electrons and carbon in engineered *Synechocystis* strains by the inactivation of flavodiiron protein Flv1/3. Natively, this heterodimer plays a distinct role as an alternative sink for excess electrons from the photosynthetic light reactions, and is therefore linked with the metabolic electron flux distribution that affects the ATP/NADPH ratio of the cell. From engineering viewpoint, Flv1/3 activity may thus (i) compete with downstream reactions for reducing equivalents that could potentially be rescued for the production of specific target chemicals, and/or (ii) alter the intracellular redox equilibria to favor certain pathways (i.e. end-products) over others.

With the objective to evaluate the effect of *flv3* deletion on the metabolic flux distribution and efficiency in *Synechocystis*, a number of engineered strains excreting sucrose as the target end-product (Fig. 1) were constructed (Table 1) and characterized. As a systematic observation throughout the analyses, the oxygen photoreduction activity of the strains harboring the native *flv3* appeared to be exceptionally high in the cells grown under 1% CO_2_ and osmotic stress. The light-induced oxygen uptake reached up to 50% of the gross photosynthetic O_2_ production as measured under high irradiancies by MIMS (Figs. 2c, 3c, 4c, 5c; red vs. black line). However, in the *flv3* deletion strains, this activity was effectively reduced or lost, and the cells exhibited only residual O_2_ uptake (Figs. 2d, 3d, 4d, 5d; red line) in comparison to the Flv1/3 control strains. While this residual oxygen uptake was expected to result, at least in part, from increased respiratory glycolytic metabolism associated with observed sucrose uptake and utilization in the Δ*flv3* strains, there was no indication of any significant activity of other alternative native electron acceptors (like Cyd/Cox [17] or Flv2/4 [9]) under any of the conditions tested. This, together with restoration of oxygen uptake resulting from Flv3 over-expression in the Δ*flv3* background (Additional file 1: Fig. S4b), implicated that the Mehler-like reaction catalyzed by the Flv1/3 heterodimer [8, 9, 12] was the primary reason for the recorded O_2_ consumption. Thus, the electrons normally consumed in by Flv1/3 could be available and potentially provide a biosynthetic advantage for the Δ*flv3* mutant, especially as the fitness of the deletion strains appeared not to be compromised based on growth (Figs. 2b, 3b, 4b, 5b) (Additional file 1: Fig. S2b). This was corroborated by the slight yet consistent increase in the total carbon uptake of the Δ*flv3* mutant strains observed in MIMS (Figs. 2c, d, 3c, d, 4c, d, 5c, d; blue line) (Additional file 1: Table S1), suggesting that the deletion of *flv3* could under certain conditions be beneficial in respect to carbon fixation in comparison to the strains with the native Flv3 background. The enhancement of CO_2_ fixation has also been reported earlier in engineered cyanobacteria, and linked with possible sink-effect where enhanced product formation creates a pull that upregulates the upstream photosynthetic reactions [14, 18]. In our case, however, the PSII turnover (measured as the gross O_2_ evolution) was significantly downregulated in the Δ*flv3* strains under all the test conditions (Figs. 2c, d, 3c, d, 4c, d, 5c, d; black line). Together the findings indicated that (i) the Δ*flv3* mutant strains exhibited lower total photosynthetic efficiency, but (ii) allocated a higher proportion of the resources downstream PSI for the biosynthesis of organic compounds as compared to the control strains with the native Flv1/3. In another words, although the overall photosynthetic efficiency was reduced, a higher total amount of energy could be funneled into productive use in the Δ*flv3* strains.

In this work we evaluated the effect of Flv1/3 inactivation on carbon distribution in *Synechocystis* in respect to three distinct end-products, sucrose generated via two engineered pathway variations, and the endogenous storage compounds PHB and glycogen. Clearly, the most significant benefit of *flv3* deletion was observed in the production of PHB in Δ*flv3* cells cultivated under high light 200 μmol photons m^−2^ s^−1^ under 1% CO_2_ atmosphere (Additional file 1: Fig. S5). Under these conditions PHB was the primary sink over sucrose and glycogen in the *flv3* deletion strains (Table 1; strains S01:Δ*flv3* and S02:Δ*flv3*), with the total amount exceeding two-fold in comparison to the corresponding control strains **(**Table 1; strains S01 and S02) (Fig. 6). This demonstrates that the deletion of *flv3* provides means for improving photoautotrophic production of PHB in *Synechocystis*, but could also be directly implemented for enhancing the flux towards 3-hydroxybutyrate [19]—an industrially relevant monomeric derivative of PHB that is spontaneously excreted out from the cell, and could be used for development of a continuous production system. The inactivation of Flv3 was also shown to improve sucrose production in the strain expressing the heterologous sucrose permease CscB (Table 1; strain S01:Δ*flv3*) under low or moderate light (Fig. 2a) (Additional file 1: Fig. S2a), but generally the absolute productivities were very low (Fig. 6). Notably, additional genetic modifications (i.e. *sps* over-expression and *ggpS* deletion in S02) that were initially introduced to reinforce the sucrose pathway (Table 1; strains S02) in fact decreased sucrose productivity of the Δ*flv3* over the control strains—showing that sucrose is not the optimal end-product for the Flv1/3-deficient strains under the experimental set-up. The production of glycogen was enhanced in the S02:Δ*flv3* strain grown under 50 μmol photons m^−2^ s^−1^, but in regards to the total carbon flux, the impact of *flv3* deletion was marginal (Fig. 6).

The results implicitly demonstrate that the deletion of *flv3* drastically modulates the electron flux distribution and carbon allocation in engineered *Synechocystis* cells. At the same time, the findings support the conception that the inactivation of alternative photosynthetic electron acceptors, such as Flv3, could potentially be used for enhancing specific downstream metabolic reactions. However, the flux ratios between the alternative electron sinks, as here represented by sucrose, PHB and glycogen (Fig. 6), are determined by environmental conditions such as the light intensity and the genetic layout of the associated pathways. Thus, targeting specific pathways of interest, requires comprehensive understanding of the complex endogenous regulatory networks and interlinked factors, which define the most favorable path for photosynthetic water-derived electrons under alternative metabolic environments. In the present work, the increased electron pressure and related changes in cellular redox balance that result from *flv3* deletion—together with simultaneous sucrose production—are likely to interfere with the native mechanisms that regulate the accumulation and use of PHB and glycogen in *Synechocystis* [20, 21]. One of the underlying causes for the observed effects is expected to be the ATP/NADPH ratio in the Δ*flv3* cells, as many downstream processes and associated control circuits are affected by this homeostasis [22].

We have shown that the transfer of electrons by Flv1/3 to O_2_ constitutes a significant share of the total photosynthetic electron flux under various conditions, especially under high light (Figs. 2c, d, 3c, d, 4c, d, 5c, d; red line). This alternative electron transfer is coupled to ATP production, while limiting the amount of electrons that are used for the reduction of NADP^+^ to NADPH. Thus when functional, Flv1/3 contributes to the increase of the relative intracellular ATP/NADPH ratio via the native Mehler-like oxygen photoreduction. In reverse, the inactivation of Flv1/3 would be expected to increase the relative levels of NADPH, with a consequent reduction in the ATP/NADPH ratio. This condition could become especially relevant in the Δ*flv3* cells under high light cultivation, as demonstrated by the decrease of the ATP/NADPH ratio of the deletion strain grown at 200 μmol photons m^−2^ s^−1^ in comparison to WT (Fig. 7). Despite the fact that the total carbon fixation was shown to be slightly increased in the Δ*flv3* strains (Figs. 2c, d, 3c, d, 4c, d, 5c, d; blue line), this may pose a problem with many downstream biosynthetic processes that require higher ATP/NADPH ratios than primary CO_2_ fixation. These pathways may consequently become limited by inadequate ATP, and result in metabolic responses such as the observed downregulation of the photosynthetic machinery that could compromise the overall production system efficiency. At the same time, such change in the metabolic redox state could be in the favor of pathways that function to counterbalance the effect, and compensate for the relative intracellular ATP deficiency. This is supported by the significant enhancement of PHB production in the Δ*flv3* mutant, as the biosynthesis of PHB is not limited by the redox poise of the cell, and specifically increases the ATP/NADPH ratio that is reduced by the deletion of *flv3*. The observation is consistent with the native redox control of PHB biosynthesis, which is induced under nitrogen limitation [23, 24] that shifts the balance between NADP^+^ and NADPH towards the reduced form (i.e. decreases the ATP/NADPH ratio) [23]. Under these conditions PHB serves as a redox sink to use excess NADPH in the cell [23], in analogy what is observed for the ∆*flv3* strain. Several computational analyses on engineered pathway fluxes in cyanobacteria have predicted analogous interconnections between the intracellular redox state and the target pathways. For example, the production of 1-octanol through an engineered NADPH-dependent pathway is expected to be enhanced by the inactivation of Flv1/3, that potentially increases the relative abundance of NADPH [18]. In a similar manner, stoichiometric network analysis has suggested that target pathways that have lower relative demand for ATP over NADPH than generally required for cell growth, can be favoured on the expence of biomass accumulation by genetic modifications that reduce the intracellular ATP/NADPH ratio [25]. In the case of the ∆*flv3* mutant, the observed upregulation of PHB biosynthesis is likely to be further interlinked with other reactions in the central carbon metabolism, as PHB is produced from the intracellular glycogen carbon pool [24], that may also affect the use of other available carbohydrate substrates. In line with this, we also observed significant increase in the sucrose uptake (Figs. 3a, 4a; red line) and respiratory metabolism (Figs. 3d vs. 2d, 4d vs 5d; red line) in the Δ*flv3* strains under the same conditions that promoted PHB accumulation. Sucrose consumption has been previously reported for engineered *Synechocystis* strains that excrete sucrose [26], but the enhancement of the effect in the Δ*flv3* mutant over the control strains may reflect metabolic acclimation to re-establish the native ATP/NADPH equilibrium in the absence of Flv1/3. This would take place through upregulated aerobic carbohydrate breakdown and cellular respiration which ultimately produces ATP, using sucrose that has been photosynthetically produced and secreted into the medium by the same cells.

The founding idea in this work was to evaluate the possible biosynthetic advantage of disabling the Mehler-like activity catalyzed by the alternative electron acceptor Flv1/3, while recognizing that the function is important for protecting the cell against fluctuations in the photosynthetic redox poise. Flv1/3 has been shown to be essential for cyanobacteria specifically under fluctuating light [9, 11, 12], implying that biotechnological applications that use natural light would not be feasible for ∆*flv3* strains, unless introduced product pathways could provide sufficiently efficient redox sinks to rescue the phenotype. As observed before [9, 12] and demonstrated in this study, however, the lack of Flv1/3 appears not to be critical under constant illumination, so the strategy could be applicable in strain engineering under controlled light systems. The metabolic effects associated with flavodiiron proteins are also linked with carbon availability [9, 12], and while the limitation of CO_2_ and consequent down-regulation of CBB cycle generally increases the need for alternative electron acceptors to dissipate excess electrons, different enzymes function under distinct conditions [12]. As Flv1/3 is expressed and active also when CO_2_ is abundant, and serves as the main alternative electron sink responsible for oxygen photoreduction in *Synechocystis* under high carbon concentrations [12], it is a prominent deletion target specifically for applications that rely on additional supplemented CO_2_. In the cultivation setup used in this work, the *flv3* deletion strain appears not to be critically damaged by light stress under constant illumination, while the elevated CO_2_ level (1%) is sufficiently high to avoid overlap with other native alternative electron transfer routes (Flv2/4, NDH-1) which are active under atmospheric carbon concentrations [12].

Finally, although most of the observed effects resulting from *flv3* deletion are directly or indirectly associated with the Mehler-like oxygen photoreduction activity of Flv1/3, it is still unclear whether or when the lack of the homo-oligomeric form Flv3/3 also contributes to the outcome. Over-expression of Flv3 has been previously shown to partially rescue the impaired growth of ∆*flv1* strain (devoid of Flv1/3 activity) under fluctuating light conditions [9], but the reaction is oxygen-independent and thus clearly distinct from the function of Flv1/3. As the biological role, associated reactions and interaction partners of Flv3/3 have not yet been elucidated, it is not known if the enzyme is involved in functions that affect electron partitioning towards different metabolic pathways under constant light.

## Conclusions

The work presented here demonstrates the effects of the inactivation of the alternative electron acceptor Flv1/3 in engineered *Synechocystis* strains on the production of sucrose, PHB and glycogen, and associated photosynthetic gas fluxes under various light conditions. Besides assisting further design of the specified target pathways, the findings can directly be applied to enhance the photoautotrophic production of associated compounds such as the monomeric PHB derivative 3-hydroxybutyrate [19, 27]. In a broader view, the results provide a generic perspective for optimizing the electron flux between photosynthetic light reactions and the downstream biosynthetic pathways, with the objective to funnel cellular resources more effectively towards the generation of target chemicals. The work also underlines the potential effects of the changes in the ATP/NADPH ratio whenever alternative electron acceptors are inactivated, which appears to be an important factor in determining carbon partitioning in engineered cyanobacteria.

## Materials and methods

### Reagents and enzymes

The enzymes used in the work were purchased from New England BioLabs (US) or from Thermo Fisher Scientific (US), and the commercial kits from manufactures as described below. The oligonucleotides were ordered from Eurofins MWG Operon (DE), and larger gene fragments from GenScript (US). All chemicals used in this study were purchased from Sigma-Aldrich (US) unless mentioned otherwise.

### Microbial strains and standard culture conditions

A glucose-tolerant substrain of *Synechocystis* sp. PCC 6803 and the corresponding Δ*flv3* strain, kindly provided by Professor Aaron Kaplan [10],  were used for generating all the cyanobacterial strains in this study (Table 1). The cells were grown in liquid BG-11 medium buffered with 20 mM TES-KOH (pH 8.0) [28] with supplemented 20 μg ml^−1^ spectinomycin (Sp), 5–8 μg ml^−1^ chloramphenicol (Cm) (for strains harboring the replicative pDF plasmid) and 20 µg/ml kanamycin (Km) (for genomic integration constructs until segregation was confirmed). The liquid cultures were typically carried out in 250 ml Erlenmeyer flasks (100 ml medium) incubated at 30 °C in ~ 120 rpm orbital shaking under continuous light of 20–200 μmol photons m^−2^ s^−1^ under 1% CO_2_ atmosphere (MLR-351 growth chamber; Sanyo, JP or Algaetron 230 growth chamber; Photon Systems Instruments, CZ). Solid plate cultivations were conducted on BG-11 plates containing additional 1.5% (w/v) Bactoagar (Difco, US) and 0.3% (w/v) sodium thiosulfate under continuous light of 50 μmol photons m^−2^ s^−1^ under 1% CO_2_ atmosphere (MLR-351).

*Escherichia coli* strain DH5α was used as a generic host for plasmid propagation. The cells were grown in Luria–Bertani (LB) medium at 37 °C in a shaker at 150–200 rpm or on the solid LB plates containing 1.5% (w/v) agar. LB medium was supplemented with appropriate antibiotics (100 µg ml^−1^ ampicillin, 50 µg ml^−1^ Sp and 34 µg ml^−1^ Cm.

### Molecular biology and plasmid construction

The expression constructs (Additional file 1: Table S3) were assembled by using standard molecular biology techniques and commercial extraction kits (Qiagen, DE), following a modular cloning strategy described earlier [29]. The target genes (*cscB*, *flv3*, *sps*) were ordered as synthetic DNA fragments (GenScript, US), and expressed in *Synechocystis* from an autonomously replicating pDF-lac2 plasmid [29] under the isopropyl β-D-1-thiogalactopyranoside (IPTG) -inducible promoter P_A1lacO-1_ and RBS from *Synechocystis cpcB*. The *ggpS* (*sll1566*) gene knock-out was generated by replacing the target gene with  a kanamycin resistance cassette by homologous recombination. The homologous flanking regions were PCR-amplified from *Synechocystis* chromosomal DNA, and the kanamycin cassette from pCOLADuet-1 (Novagen), using the corresponding primers listed in Additional file 1: Table S4. The backbone vector for the inserts (see restriction sites in Additional file 1: Table S4) was generated by inserting the following NdeI-HindIII fragment into the commercial vector pUC57: 5′-CATATGTTTTCTAGAATTGGATCCAAACCCGGGTTCACCTCGAGAAATTTATCAAAAAGAGTGTTGACTTGTGAGCGGATAACAATGATACTTAGATTCAATTGTGAGCGGATAACAATTTCACACAGAATTCATTAAAGACTAGTAAGGTACCCGGTGTCGACTTTCGGCCGATACCATGGTTAGATATCTAAGCTAACTTTAATAAAACGAAAGGCTCAGTCGAAAGACTGGGCCTTTCGTTTTATCTGTTGTTTGTAGTACTAGATAACTTAGATAAGGCCATCCTGACGGATGGCCTTTTTGCGTTTCTACCTAGGATAAGATCTCATATCGATAACAAGCTT-3′.

### *Synechocystis* transformation and recombinant strain verification

*Synechocystis* cells were transformed at the logarithmic growth phase with ~ 2 μg plasmid DNA via natural transformation [9]. The transformants were selected on BG-11 agar plates containing 2 μg ml^−1^ Cm and 4 μg ml^−1^ Sp (pDF-derived constructs) or 20 µg ml^−1^ Km (for pUC57-derived constructs), and re-streaked on new plates with increasing antibiotic concentrations. The presence of replicative plasmids and the full segregation of the *ggpS* and *flv3* deletions in the cells were verified by amplification of DNA by a colony PCR (Additional file 1: Fig. S1) using primers described in Additional file 1: Table S3.

### Spectrophotometric characterization

Growth of the *Synechocystis* strains was monitored by measuring the increase of culture optical density at 750 nm (OD_750nm_) using GENESYS 10S UV–Vis spectrophotometer (Thermo Fisher Scientific, US). Pigment analysis (Additional file 1: Fig. S3) was carried out with Olis CLARiTY 14 dual beam spectrophotometer equipped with 8 ml ICAM cuvette (On Line Instrument Systems, Inc., US). Raw absorption values (AU) of the scans (370–700 nm) were converted to real absorbance values (AU/cm) using Fry’s method [14].

### Quantitative analysis of sucrose, PHB and glycogen production

For quantitating sucrose, PHB and glycogen, the generated *Synechocystis* strains were grown under the standard conditions (under 50 µmol photons m^−2^ s^−1^ continuous light) to OD_750 nm_ ~ 2. The precultures were then diluted to OD_750 nm_ ~ 0.4, divided into four independent replicates, and allowed to adapt for 24 h to 400 mM NaCl under the specified light conditions (20, 50 or 200 µmol photons m^−2^ s^−1^). After the adaptation period, the OD_750 nm_ was adjusted to ~ 0.5 by gently pelleting and re-suspending the cells into 100 ml of fresh BG-11 supplemented with 400 mM NaCl and 1 mM IPTG. At the indicated time points (see “Results” section), 1 ml samples were collected and stored at − 80 °C until analysis. Analysis of the target products was performed with commercial analytical kits according to the manufacturers’ instructions, carried out in 96-well plate format using a microplate reader (Infinite M200 PRO, Tecan, CH). The kits for measuring sucrose (Sucrose/d-Glucose Assay Kit; Megazyme, US) and glycogen (Total Starch Assay Kit; Megazyme, US) were based on spectrophotometric quantitation of d-glucose released from enzymatic breakdown of sucrose in the supernatant (by β-fructosidase) or intracellular glycogen (by α-amylase and amyloglucosidase), respectively, with a coupled glucose oxidase/peroxidase GOPOD assay (510 nm). Analysis of PHB (D-3-Hydroxybutyric Acid Assay Kit; Megazyme, US) was based on alkaline lysis of intracellular PHB into D-3-hydroxybutyric acid, followed by an enzymatic reaction to produce a stoichiometric amount of NADH (by 3-hydroxybutyrate dehydrogenase), coupled to a subsequent NADPH-dependent diaphorase reaction to generate a colored end-product (492 nm).

### Cellular gas flux analysis

Membrane inlet mass spectrometry (MIMS) (see [30]) was used to measure the steady state cellular gas flux rates (uptake of O_2_ and CO_2_, and O_2_ evolution) directly from cell suspensions under increasing light intensities of 0, 25, 50, 75, 100, 200 and 500 μmol photons m^−2^ s^−1^. The system consisted of a cuvette in which the sample chamber (under atmospheric pressure) was separated by a Teflon membrane (Hansatech Instruments, UK) from the high vacuum line of Sentinel PRO magnetic sector mass spectrometer (Thermo Fisher Scientific, US), and a remote halogen lamp (Dolan-Jenner Industries, US) unit calibrated to the required irradiances (Li-250A, LI-COR instruments, US). The samples were collected from four replicate cultures at day 5 after induction, washed once, and resuspended in fresh modified growth medium (nitrate free BG-11 buffered to pH 8.0 with 50 mM HEPES, containing 400 mM NaCl,) to 10 µg ml^−1^ Chl *a*. After loading, the samples were briefly purged with N_2_ gas to reduce levels of solubilized ^16^O_2_, supplemented with 50 µg ml^−1^ bovine carbonic anhydrase (Sigma-Aldrich, US) and 1 mM HCO_3_^−^ (Merck, DE), and enriched in ^18^O_2_ (CK Isotopes, UK) by introducing a 50 µl bubble in the cuvette for several minutes in darkness. The analysis was carried out by scanning ion masses m/z 32, 36 and 44 corresponding to ^16^O_2_, ^18^O_2_ and CO_2_, respectively, at a ten second cycles. Final Chl *a* concentration, determined spectrophotometrically in 90% methanol according to Porra et al. [12], was conducted at completion of each measurement for standardizing the calculated gas exchange rates. Instrument calibrations and gas consumption rates were corrected as per methods outlined in Beckmann et al. [13]. Average rates at each irradiance were calculated in Origin 2015, IBM SPSS Statistics 24 (see Figs. 2, 3, 4 5) (Additional file 1: Fig. S4), and used for pairwise statistical comparison (*t* test) between the Δ*flv3* strains and the corresponding controls (see Additional file 1: Table S1).

### In silico analysis of the ATP/NADPH demand between the alternative pathways

The *Synechocystis* metabolic model iSynCJ816 [16] was used as the basis for estimating the relative differences in the net ATP and NADPH demand between the alternative pathways towards sucrose, PHB and glycogen (Fig. 1). First, the enzymatic steps from the common cellular intermediate glyceraldehyde-3-phosphate (G3P) towards the three alternative products were selected (see Additional file 1: Table S2), followed by the calculation of the net consumption/production of ATP and NADPH in the successive catalytic steps within the pathways. The calculations took into account the number of molecules of ADP/ATP and NADP^+^/NADPH generated in each reaction, and the number of times each reaction is required for the production of one target molecule (or the production and incorporation of one immediate precursor, 3-hydroxybutyryl-CoE or UDP-glucose, for the polymeric PHB and glycogen, respectively). Also the reactions branching from the primary pathways were considered whenever the reactions were unambiguous in the model (i.e. only a single reaction in the model produces the molecule).

### Quantitation of ATP and NADPH in *Synechocystis* wild-type and Δ*flv3* cells

For the analysis of ATP and NADPH, *Synechocystis* wild-type and Δ*flv3* strains were grown in 100 ml liquid cultures in 250 ml Erlenmeyer flasks in BG-11 medium buffered with 20 mM TES-KOH (pH 8.0). Cultures were diluted to OD_750nm_ of 0.1 and incubated at 30 °C in ~ 120 rpm orbital shaking under continuous light of 200 μmol photons m^−2^ s^−1^ and 1% CO_2_ atmosphere. For each measurement point, 10 ml samples were taken from three independent cultures on days 1 and 3 (Additional file 1: Fig. S7), frozen in liquid nitrogen and stored at − 80 °C until analysis.

The cells were thawed, pelleted (4696 g, 10 min, 4 °C) and re-suspended in 1:1 mixture of Phosphate Buffered Saline, pH 8.0 and 0.2 N NaOH supplemented with 1% (w/V) dodecyltrimethylammonium bromide (DTAB), followed by lysis in the presence of ~ 250 mg Zirconium Oxide Beads (0.15 mm, Next Advance, US) using Bullet Blender Storm 24 (2 × 5 min, speed 12, Next Advance, US). The resulting supernatant (500 µl) was analyzed in three technical replicates using commercial analytical kits for ATP (ADP/ATP Ratio Assay Kit, Sigma-Aldrich, US) and NADPH (NADP/NADPH-Glo™ Assay, Promega, US) according to manufacturer’s instructions. Both kits are based on the ATP-consuming luciferase reaction, which in the NADPH kit is coupled to NADPH-dependent reaction producing the substrate luciferin. The resulting bioluminescence was recorded using a microplate reader (Infinite M200 PRO), and used for calculating the signals as Relative Luminescence Units (RLU) per volume per cell density.

## Supplementary information

**Additional file 1: Fig. S1.** Colony PCR verification of the engineered *Synechocystis* strains generated in this study. **Fig. S2.** Characterization of the engineered *Synechocystis* strains S01 and S01:Δ*flv3* grown under continuous 20 μmol photons m^−2^ s^−1^ light. **Fig. S3.** Spectrophotometric analysis of pigments in the engineered sucrose-producing *Synechocystis* strains grown under 50 and 200 μmol photons m^−2^ s^−1^ light, measured on days 2, 5 and 10. **Fig. S4.** Partial complementation of Flv3 inactivation in engineered *Synechocystis* Δ*flv3* strain grown under continuous light of 50 μmol photons m^−2^ s^−1^. **Fig. S5.** Quantitative analysis of polyhydroxybutyrate content in the engineered *Synechocystis* strains grown for 12 days under continuous 50 and 200 μmol photons m^−2^ s^−1^ light. **Fig. S6.** Quantitative analysis of glycogen content in engineered sucrose-producing *Synechocystis* strains at different time points (0**–**12 days), grown under continuous 50 and 200 μmol photons m^−2^ s^−1^ light. **Fig. S7.** Growth curve of *Synechocystis* wild-type and ∆*flv3* strains grown under 200 μmol photons m^−2^ s^−1^ light for 24 h and 72 h. **Fig. S8.** Quantitation of the relative ATP and NADPH content of *Synechocystis* wild-type and ∆*flv3* strains grown under 200 μmol photons m^−2^ s^−1^ light for 24 h and 7 2h. **Table S1.** Summary of the calculated significances for the cellular gas fluxes measured by MIMS (Figs. 2, 3, 4, 5) for the *Synechocystis* strains generated in this study. **Table S2.** Simplified list of enzymatic reactions towards sucrose, PHB and glycogen in *Synechocystis* from the common metabolic intermediate glyceraldehyde-3-phosphate, and estimation of relative ATP/NADPH demand between the pathways. **Table S3.** List of plasmids generated and used in this study. **Table S4.** List of PCR primers used in this study.

## Data Availability

The datasets used and/or analysed during the current study are available from the corresponding author on reasonable request
