# Supplementary material for: Redirecting photosynthetic electron flux in the cyanobacterium Synechocystis sp. PCC 6803 by the deletion of flavodiiron protein Flv3
Source: Microb Cell Fact. 2019 Nov 5;18:189. doi: 10.1186/s12934-019-1238-2 (PMC6833302; doi:10.1186/s12934-019-1238-2)
Supplement: Supplementary file 1 — Additional file 1: Fig. S1. Colony PCR verification of the engineered Synechocystis strains generated in this study. Fig. S2. Characterization of the engineered Synechocystis strains S01 and S01:Δflv3 grown under continuous 20 μmol photons m−2 s−1 light. Fig. S3. Spectrophotometric analysis of pigments in the engineered sucrose-producing Synechocystis strains grown under 50 and 200 μmol photons m−2 s−1 light, measured on days 2, 5 and 10. Fig. S4. Partial complementation of Flv3 inactivation in engineered Synechocystis Δflv3 strain grown under continuous light of 50 μmol photons m−2 s−1. Fig. S5. Quantitative analysis of polyhydroxybutyrate content in the engineered Synechocystis strains grown for 12 days under continuous 50 and 200 μmol photons m−2 s−1 light. Fig. S6. Quantitative analysis of glycogen content in engineered sucrose-producing Synechocystis strains at different time points (0–12 days), grown under continuous 50 and 200 μmol photons m−2 s−1 light. Fig. S7. Growth curve of Synechocystis wild-type and ∆flv3 strains grown under 200 μmol photons m−2 s−1 light for 24 h and 72 h. Fig. S8. Quantitation of the relative ATP and NADPH content of Synechocystis wild-type and ∆flv3 strains grown under 200 μmol photons m−2 s−1 light for 24 h and 7 2h. Table S1. Summary of the calculated significances for the cellular gas fluxes measured by MIMS (Figs. 2, 3, 4, 5) for the Synechocystis strains generated in this study. Table S2. Simplified list of enzymatic reactions towards sucrose, PHB and glycogen in Synechocystis from the common metabolic intermediate glyceraldehyde-3-phosphate, and estimation of relative ATP/NADPH demand between the pathways. Table S3. List of plasmids generated and used in this study. Table S4. List of PCR primers used in this study. [file 12934_2019_1238_MOESM1_ESM.pdf]

# Additional file 1

Thiel, K., Patrikainen, P., Nagy, C., Fitzpatrick, D., Pope, N. Aro, E-M., and Kallio, P*.
Redirecting photosynthetic electron flux in the cyanobacterium *Synechocystis* sp. PCC 6803 by the deletion of flavodiiron protein Flv3

* Corresponding author. Email: pataka@utu.fi

**This file includes:**

**Fig. S1.** Colony PCR verification of the engineered *Synechocystis* strains generated in this study. Page 2.
**Fig. S2.** Characterization of the engineered *Synechocystis* strains S01 and S01:Δ*flv3* grown under continuous 20 µmol photons $m^{-2}$ $s^{-1}$ light. Page 3.
**Fig. S3.** Spectrophotometric analysis of pigments in the engineered sucrose-producing *Synechocystis* strains grown under 50 and 200 µmol photons $m^{-2}$ $s^{-1}$ light, measured on days 2, 5 and 10. Page 4.
**Fig. S4.** Partial complementation of Flv3 inactivation in engineered *Synechocystis* Δ*flv3* strain grown under continuous light of 50 µmol photons $m^{-2}$ $s^{-1}$. Page 5.
**Fig. S5.** Quantitative analysis of polyhydroxybutyrate content in the engineered *Synechocystis* strains grown for 12 days under continuous 50 and 200 µmol photons $m^{-2}$ $s^{-1}$ light. Page 6.
**Fig. S6.** Quantitative analysis of glycogen content in engineered sucrose-producing *Synechocystis* strains at different time-points (0-12 days), grown under continuous 50 and 200 µmol photons $m^{-2}$ $s^{-1}$ light. Page 7.
**Fig. S7.** Growth curve of *Synechocystis* wild-type and Δ*flv3* strains grown under 200 µmol photons $m^{-2}$ $s^{-1}$ light for 24h and 72h, indicating the sampling points for Fig. S8. Page 8.
**Fig. S8.** Quantitation of the relative ATP and NADPH content of *Synechocystis* wild-type and Δ*flv3* strains grown under 200 µmol photons $m^{-2}$ $s^{-1}$ light for 24h and 72h. Page 9.
**Table S1**. Summary of the calculated significances for the cellular gas fluxes measured by MIMS (**Figs. 2-5**) for the *Synechocystis* strains generated in this study. Page 10-11.
**Table S2**. Simplified list of enzymatic reactions towards sucrose, PHB and glycogen in *Synechocystis* from the common metabolic intermediate glyceraldehyde-3-phosphate, and estimation of relative ATP/NADPH demand between the pathways. Page 12-13.
**Table S3.** List of plasmids generated and used in this study. Page 14.
**Table S4.** List of PCR primers used in this study. Page 15.

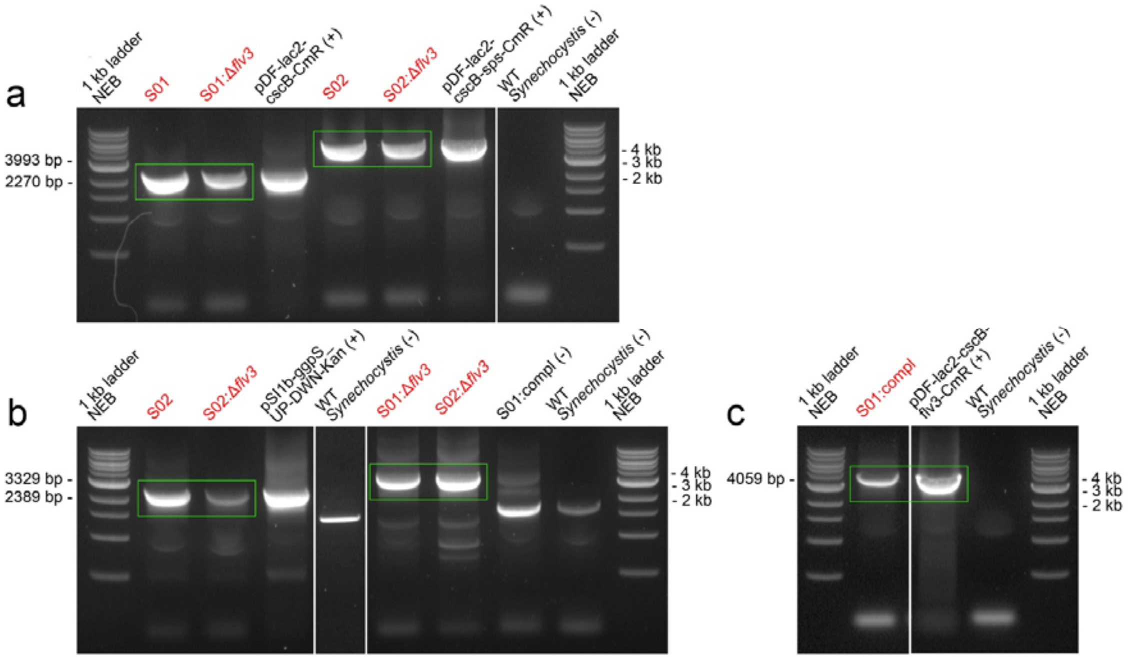


**Fig. S1.** Colony PCR verification of the *Synechocystis* strains generated in this study (red font; see Table 1) as visualized by agarose gel electrophoresis. (**a**) The presence of the expression cassette for CscB (S01; S01:Δ*flv3*) and CscB+SPS (S02; S02:Δ*flv3*) was confirmed using the corresponding plasmids as positive controls (pDF-lac2-cscB-CmR; pDF-lac2-cscB-sps-CmR), and WT *Synechocystis* as a negative control (primers in pDF-lac2_FOR and pDF-lac2_REV; see Table S4). (**b**) The deletion of *ggpS* (S02; S02:Δ*flv3*) was confirmed using the plasmid pSI1b-ggpS_UP-DWN-Kan as a positive control, and WT *Synechocystis* as a negative control (primers ggpS_UP_FOR and ggpS_DWN_REV; see Table S4). The deletion of *flv3* (S01:Δ*flv3*; S02:Δ*flv3*) was confirmed using the Flv3 complementation strain (S01:compl), and WT *Synechocystis* controls (primers flv3_FOR and flv3_REV; see Table S4). (**c**) The presence of CscB+Flv3 overexpression plasmid (S01:compl lane 2) was confirmed by using pDF-lac2-cscB-flv3-CmR as a positive control and wild-type *Synechocystis* as a negative control (primers pDF-lac2_FOR and pDF-lac2_REV; see Table S4). The expected sizes for the PCR products are shown on the left, and the correct bands have been highlighted in green.

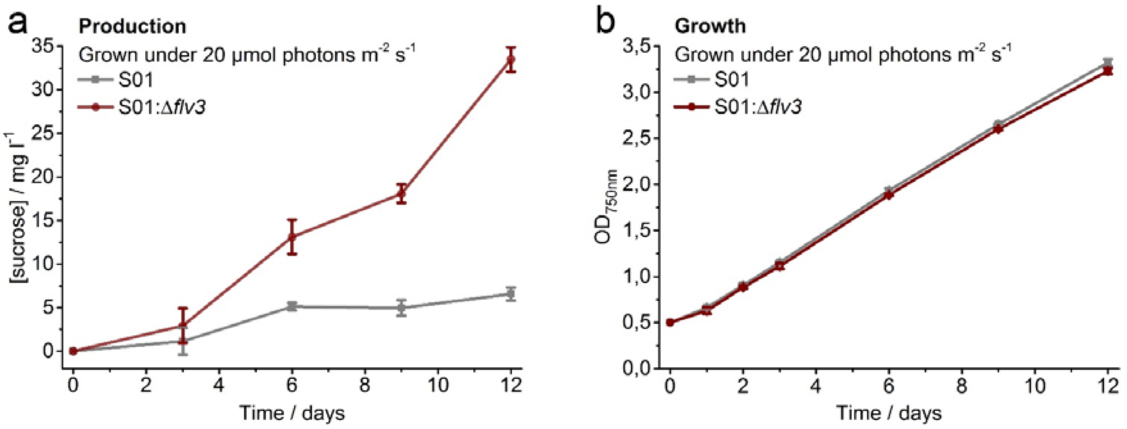


**Fig. S2.** Characterization of engineered *Synechocystis* strains grown under continuous light of 20 µmol photons $m^{-2}$ $s^{-1}$. The strains S01 (over-expression of sucrose permease CscB) and S01:Δ*flv3* (over-expression of CscB and inactivation of flavodiiron protein Flv3) were evaluated in respect to (**a**) sucrose production (mg $l^{-1}$) and (**b**) growth ($OD_{750nm}$). The control strain S01 is shown in grey and the strain S01:Δ*flv3* in brown. The strains were cultivated in 1 % $CO_2$ in the presence of 400 mM supplemented NaCl. In each case, the average and standard deviation were calculated from three to four independent experiments.

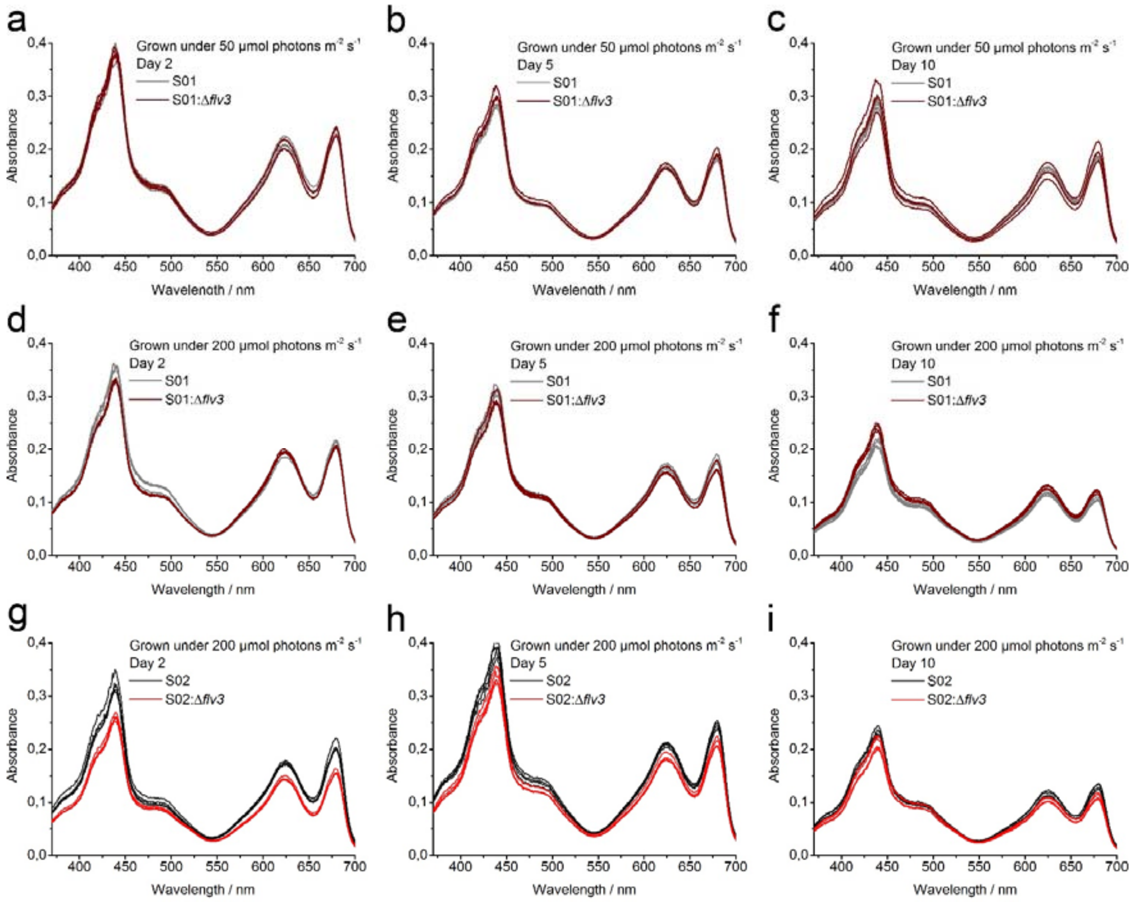


**Fig. S3.** Spectrophotometric analysis of pigments present in engineered *Synechocystis* strains (Table 1) grown under different light conditions. Absorption spectra (370 nm – 700 nm) of the strains S01 (over-expression of sucrose permease CscB) (grey) and S01:Δ*flv3* (over-expression of CscB and inactivation of flavodiiron protein Flv3) (brown) grown for (**a**, **d**) two days (**b**, **e**) five days and (**c**, **f**) 10 days under continuous growth light (50 and 200 µmol photons $m^{-2}$ $s^{-1}$, respectively). (**g**, **h**, **i**) Corresponding absorption spectra of the strains S02 (over-expression of CscB and sucrose phosphate synthase SPS, and inactivation of glucosylglyserolphosphate synthase GGPS) (black) and S02:Δ*flv3* (over-expression of CscB and SPS, and inactivation of GGPS and Flv3) (red) grown under continuous growth light of 200 µmol photons $m^{-2}$ $s^{-1}$. The strains were cultivated in 1 % $CO_2$ in the presence of 400 mM NaCl. The graphs present four independent experiments and have been normalized to 750 nm.

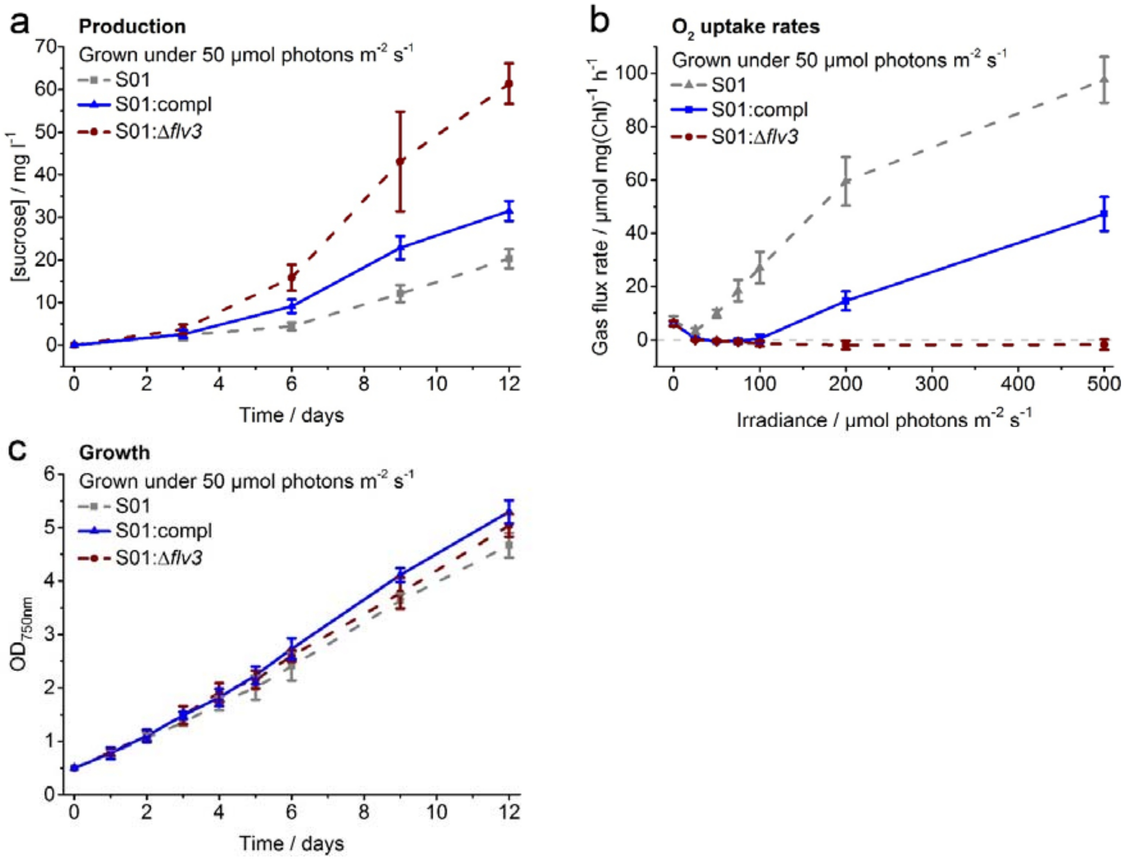


**Fig. S4.** Partial complementation of Flv3 inactivation in engineered sucrose-producing *Synechocystis* Δ*flv3* strain grown under continuous light of 50 µmol photons $m^{-2}$ $s^{-1}$. The strain S01:compl (over-expression of sucrose permease CscB and flavodiiron protein Flv3 in the Δ*flv3* background) (blue line) was evaluated in respect to (**a**) sucrose production (mg $l^{-1}$), (**b**) $O_2$ uptake measured on cultivation day 5 and (**c**) growth ($OD_{750nm}$). The strains S01 (over-expression of CscB) and S01:Δ*flv3* (over-expression of CscB and inactivation of flavodiiron protein Flv3) shown in grey and red dashed lines, respectively, have been included for comparison (see Fig. 2). The strains were cultivated in 1 % $CO_2$ in the presence of 400 mM supplemented NaCl. In each case, the average and standard deviation were calculated from three to four independent experiments.

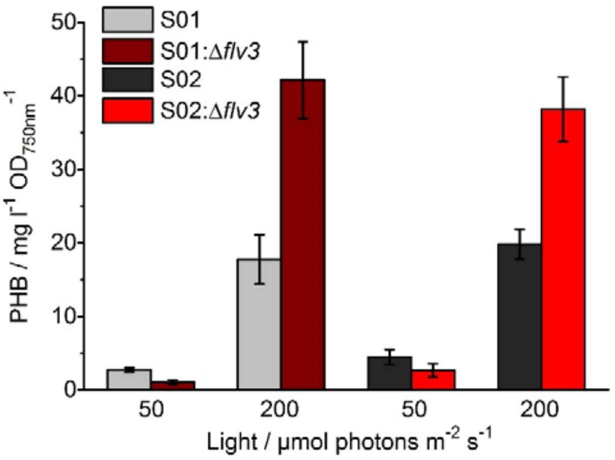


**Fig. S5.** Quantitative analysis of polyhydroxybutyrate (PHB) content in the engineered *Synechocystis* strains (Table 1) grown under different light conditions. The amount of PHB was measured from the strains S01 (over-expression of sucrose permease CscB) (grey), S01:Δ*flv3* (over-expression of CscB and inactivation of flavodiiron protein Flv3) (brown), S02 (over-expression of CscB and sucrose phosphate synthase SPS, and inactivation of glucosylglyserolphosphate synthase GGPS) (black) and S02:Δ*flv3* (over-expression of CscB and SPS, and inactivation of GGPS and Flv3) (red) grown under continuous 50 or 200 µmol photons $m^{-2}$ $s^{-1}$ light for 12 days (1 % $CO_2$ in the presence of 400 mM NaCl). PHB was broken down to 3-hydroxybutyrate and the concentration was determined colorimetrically using a commercial D-3-Hydroxybutyric Acid Assay Kit (Megazyme, US). In each case, the average and standard deviation were calculated from four independent cultivations.

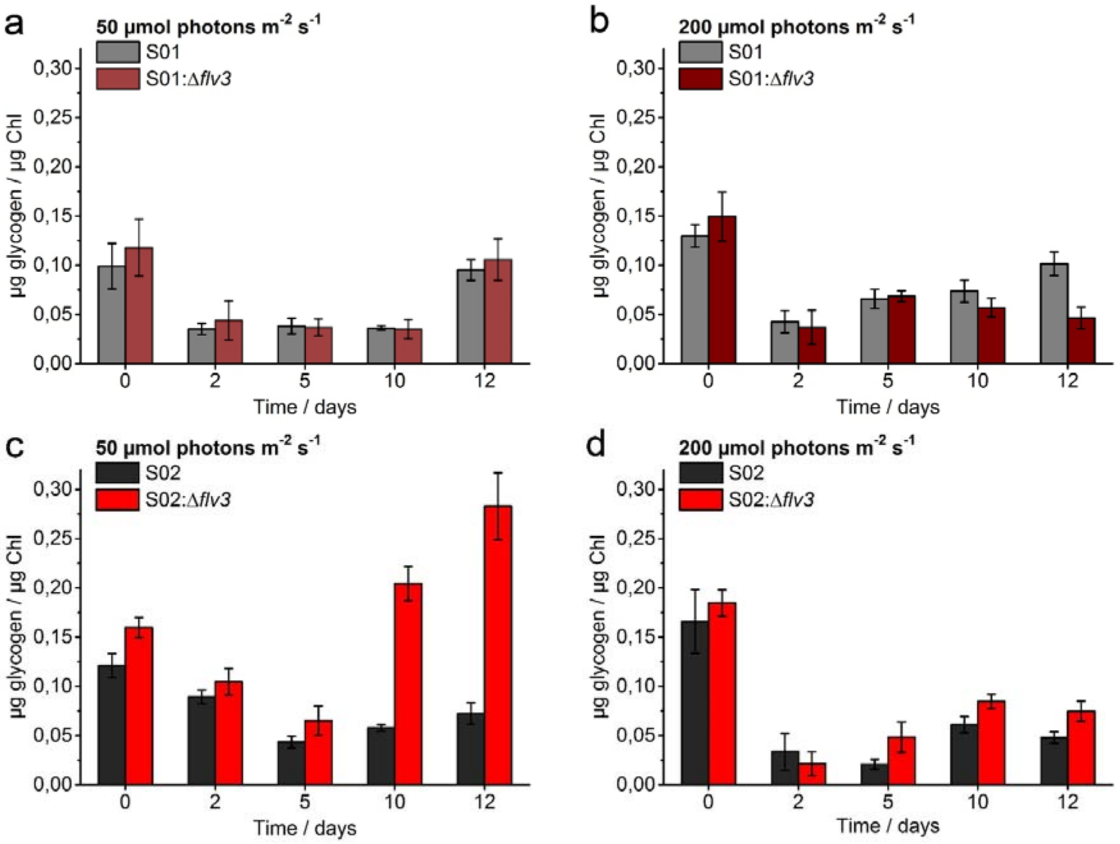


**Fig. S6.** Quantitative analysis of glycogen content at different time-points (0-12 days) in engineered *Synechocystis* strains (Table 1) grown under different light conditions. The amount of glycogen was measured from strains S01 (over-expression of sucrose permease CscB), S01:Δ*flv3* (over-expression of CscB and inactivation of flavodiiron protein Flv3), S02 (over-expression of CscB and sucrose phosphate synthase SPS, and inactivation of glucosylglyserolphosphate synthase GGPS) and S02:Δ*flv3* (over-expression of CscB and SPS, and inactivation of GGPS and Flv3) grown under continuous growth light of **(a, c)** 50 and **(b, d)** 200 µmol photons $m^{-2}$ $s^{-1}$ (1 % $CO_2$ in the presence of 400 mM NaCl). In **a** and **b**, the control strain S01 is shown in grey and the strain S01:Δ*flv3* in brown and, in **c** and **d**, the control strain S02 is shown in black and the strain S02:Δ*flv3* in red. Glycogen was broken to glucose and analyzed using a commercial Sucrose/D-Glucose Assay Kit (Megazyme, US). In each case, the average and standard deviation were calculated from four independent cultivations, and normalized to chlorophyll *a* content.

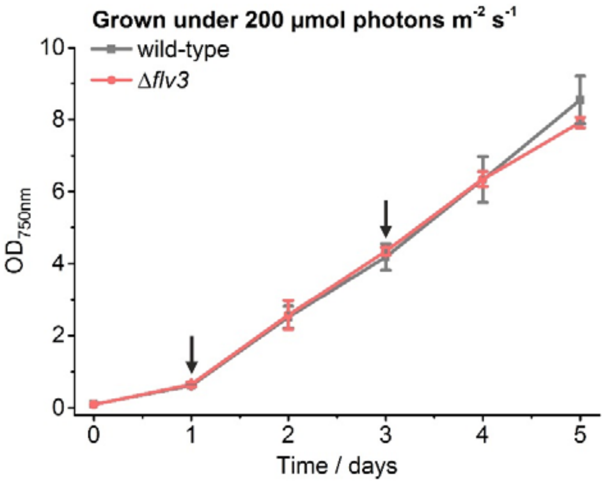


**Fig. S7.** Growth ($OD_{750nm}$) of *Synechocystis* wild-type and Δ*flv3* strains cultivated under 200 µmol photons m–2 s–1 continuous light. The samples for ATP and NADPH analysis (see Fig. S8) were taken on days 1 and 3, as indicated by black arrows. The average and standard deviation for each time-point were calculated from four independent cultures.

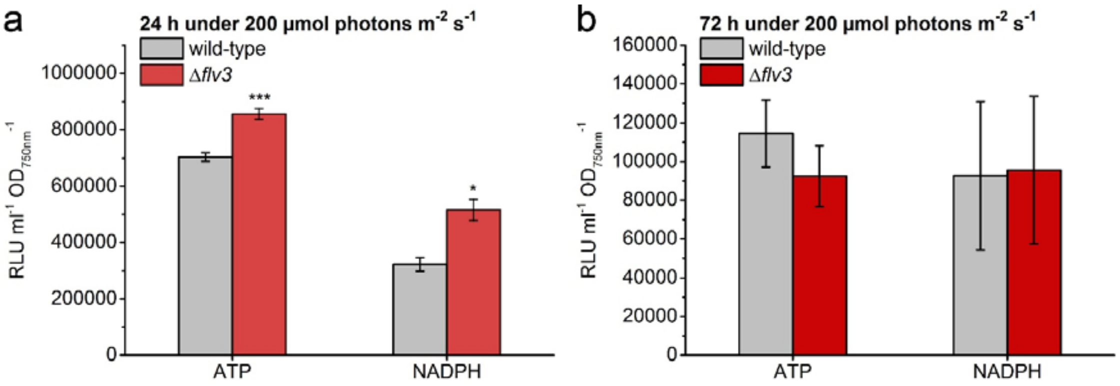


**Fig. S8:** ATP and NADPH content of *Synechocystis* wild-type and Δ*flv3* strains after cultivation under continuous 200 µmol photons $m^{-2}$ $s^{-1}$ light for (**a**) 24h and for (**b**) 72h. The values are presented in Relative Luminescence Units (RLU) per ml of culture per $OD_{750nm}$, the averages and standard deviations representing three independent cultures with three technical replicates each (n = 9). The statistical difference (*t*-test) between the strains is denoted as three ($p < 0.01$) or one ($p < 0.05$) asterisk above the Δ*flv3* error bar.

**Table S1.** Summary of the calculated significances for the cellular gas fluxes measured by MIMS (see Figs. 2-5) for the *Synechocystis* strains generated in this study. The significances represent pairwise *t*-test comparisons of each of the measurement points between a specified Δ*flv3* strain and the corresponding control strain using four independent replicates (n = 4). Statistical difference is shown in green background, and the direction of the change is indicated by + (increased value in Δ*flv3* strain in comparison to the control) and - (reduced value in Δ*flv3* strain in comparison to the control). Three signs correspond to $p < 0.01$, two signs $p < 0.02$ and one sign $p < 0.05$. Results found not to be statistically significant are presented in grey background.

| Strains / Figure ref. | Growth light intensity / µmol photons $m^{-2}$ $s^{-1}$ | | **Irradiance in MIMS / µmol photons $m^{-2}$ $s^{-1}$** | | | | | | |
|---|---|---|---|---|---|---|---|---|---|
| | | | **0** | **25** | **50** | **75** | **100** | **200** | **500** |
| S01 and S01:Δ*flv3* (see Fig. 2) | 50 | Gross $O_2$ evolution | | # | # | # | # | --- | --- |
| | | $O_2$ uptake | | --- | --- | --- | --- | --- | --- |
| | # | Total C uptake | | # | # | # | ++ | +++ | +++ |
| | # | Net $O_2$ evolution | | # | # | # | +++ | +++ | +++ |
| S01 and S01:Δ*flv3* (see Fig. 3) | 200 | Gross $O_2$ evolution | | + | | – | – | – | –– |
| | | $O_2$ uptake | | +++ | | | | --- | --- |
| | # | Total C uptake | --- | --- | --- | | | | +++ |
| | # | Net $O_2$ evolution | | | – | – | | | + |
| S02 and S02:Δ*flv3* (see Fig. 4) | 200 | Gross $O_2$ evolution | | –– | --- | --- | --- | --- | --- |
| | | $O_2$ uptake | +++ | +++ | | --- | --- | --- | --- |
| | # | Total C uptake | --- | --- | --- | – | | +++ | +++ |
| | # | Net $O_2$ evolution | --- | --- | --- | –– | –– | + | +++ |

| | | | | | | | | | | | |
|---|---|---|---|---|---|---|---|---|---|---|---|
| | # | | # | | | | | | | | |
| | | | | Gross $O_2$ evolution | | --- | --- | --- | --- | --- | --- |
| S02 and S02:Δ*flv3* (see Fig. 5) | | 50 | | $O_2$ uptake | | --- | --- | --- | --- | --- | --- |
| | # | | | Total C uptake | | | | | | | |
| | # | | | Net $O_2$ evolution | | | | | | | |

**Table S2:** Simplified list of enzymatic reactions towards sucrose, PHB and glycogen in *Synechocystis* from the common metabolic intermediate glyceraldehyde-3-phosphate, and estimation of relative ATP/NADPH demand between the pathways.

| **Enzyme [EC ID]** | **ATP/NADPH produced or used** | **Reactions / target molecule** | **Net** |
|---|---|---|---|
| **Polyhydroxybutyrate; PHB** | | | |
| Glyceraldehyde-3-phosphate dehydrogenase [EC 1.2.1.59] | 0 | 2 | 0 |
| Phosphoglycerate kinase [EC 2.7.2.3] | + 1 ATP | 2 | + 2 ATP |
| Phosphoglycerate mutase [EC 5.4.2.1] | 0 | 2 | 0 |
| Enolase [EC 4.2.1.11] | 0 | 2 | 0 |
| Pyruvate kinase [EC 2.7.1.40] | + 1 ATP | 2 | + 2 ATP |
| Pyruvate dehydrogenase [EC 1.2.4.1 / 1.8.1.4 / 2.3.1.12] | 0 | 2 | 0 |
| β-ketothiolase [EC 2.3.1.9] | 0 | 1 | 0 |
| Acetoacetyl CoA reductase [EC 1.1.1.36] | - 1 NADPH | 1 | - 1 NADPH |
| PHB synthetase [EC 2.3.1.-] | 0 | x | 0 |
| | | | **Total: + 4 ATP, - 1 NADPH** |
| **Glycogen** | | | |
| D-Fructose 1-phosphate D-glyceraldehyde-3-phosphate-lyase [EC 4.1.2.13] | 0 | 1 | 0 |
| Fructose-bisphosphatase [EC 3.1.3.11] | 0 | 1 | 0 |
| Glucose-6-phosphate isomerase [EC 5.3.1.9] | 0 | 1 | 0 |
| Phosphoglucomutase [EC 5.4.2.2] | 0 | 1 | 0 |
| Glucose-1-phosphate adenylyltransferase [EC 2.7.7.27] | - 1 ATP | 1 | - 1 ATP |
| Glycogen synthase [EC 2.4.1.21] | 0 | x | 0 |
| | | | **Total: -1 ATP** |

| | | | |
|---|---|---|---|
| **Sucrose** | | | |
| D-Fructose 1-phosphate D-glyceraldehyde-3-phosphate-lyase [EC 4.1.2.13] | 0 | 2 | 0 |
| Fructose-bisphosphatase [EC 3.1.3.11] | 0 | 2 | 0 |
| Glucose-6-phosphate isomerase [EC 5.3.1.9] | 0 | 1 | 0 |
| Phosphoglucomutase [EC 5.4.2.2] | 0 | 1 | 0 |
| Glucose-1-phosphate uridylyltransferase [EC 2.7.7.9] | 0 | 1 | 0 |
| Sucrose phosphate synthase [EC 2.4.1.14] | 0 | 1 | 0 |
| Sucrose-phosphate phosphatase [EC 3.1.3.24] | 0 | 1 | 0 |
| Sucrose permease [EC 2.7.1.69] | 0 | 1 | 0 |
| | | | **Total: No net change** |

**Table S3**. Plasmids generated and used in this study.

| Plasmid name | Use or function | References |
|---|---|---|
| pDF-lac2 | *E. coli* / *Synechocystis* shuttle vector | (1) |
| pDF-lac2-cscB-CmR | Expression of CscB | This study |
| pDF-lac2-cscB-flv3-CmR | Expression of CscB and Flv3 | This study |
| pDF-lac2-cscB-sps-CmR | Expression of CscB and SPS | This study |
| pSI1b_ggpS_UP_DWN_Kan | Disruption of *Synechocystis ggpS* with $Km^R$ | This study |
| pNiv(S3) | Assembly vector carrying RBS S3 | (1) |

**Table S4.** PCR primers used in this study for amplifying the homologous recombination regions from the genomic DNA of *Synechocystis*, for amplifying the kanamycin resistance cassette from pCOLADuet-1 and for colony PCR verification of the generated strains. Restriction sites used for subsequent cloning steps are underlined, extra overhangs are in lowercase and sequences complementary to the amplified region are in uppercase.

| Primer | Sequence (5'>3') | Restriction sites |
|---|---|---|
| ggpS_UP_FOR | tatCATATGATGAATTCATCCCTTGTGATCC | NdeI |
| ggpS_UP_REV | tatCCCGGGTCGTGGACCCAAAACAATG | XmaI |
| ggpS_DWN_FOR | tatCCTAGGAACAAGATTTAGGCGGTAAGAGG | AvrII |
| ggpS_DWN_REV | tatAAGCTTGGGTAGTTCCACCGCACA | HindIII |
| KmR_FOR | tatGTCGACGCGCTAGCATGCCTATTTGT | SalI |
| KmR_ REV | tatCCATGGCTGAGCAATAACTAGCATAACCCC | NcoI |
| pDF-lac2_ FOR | GTTGACTTGTGAGCGGATAACAATGATACTTA | |
| pDF-lac2_ REV | CCGCTTCTGCGTTCTGATTTAATCTG | |
| flv3_FOR | CATATGTTCACTACCCCCCTCCCC | |
| flv3_REV | CTCGAGTTAGTAATAATTGCCGAC | |

## References

1. K. Thiel *et al.*, Translation efficiency of heterologous proteins is significantly affected by the genetic context of RBS sequences in engineered cyanobacterium Synechocystis sp. PCC 6803. *Microb Cell Fact* **17**, 34 (2018).
2. C. J. Joshi, C. A. M. Peebles, A. Prasad, Modeling and analysis of flux distribution and bioproduct formation in Synechocystis sp. PCC 6803 using a new genome-scale metabolic reconstruction. *Algal Research* **27**, 295-310 (2017).

#
